# Computational approaches to modeling dynamos in galaxies

**DOI:** 10.1007/s41115-024-00021-9

**Published:** 2024-07-02

**Authors:** Maarit J. Korpi-Lagg, Mordecai-Mark Mac Low, Frederick A. Gent

**Affiliations:** 1 Astroinformatics, Department of Computer Science, Aalto University, P.O. Box 15400, 00076 Espoo, Finland; 2 Department of Astrophysics, American Museum of Natural History, 200 Central Park West, New York, NY 10024 USA; 3 Nordic Institute for Theoretical Physics, Hannes Alfvéns väg 12, 106 91 Stockholm, Sweden; 4 Max Planck Institute for Solar System Research, Justus-von-Liebig-Weg 3, 37707 Göttingen, Germany; 5 KTH Royal Institute of Technology and Stockholm University, Hannes Alfvéns väg 12, 106 91 Stockholm, Sweden; 6 School of Mathematics, Statistics and Physics, Newcastle University, Newcastle upon Tyne, NE1 7RU UK

**Keywords:** Dynamo, Galaxy, Numerical methods, Magnetic fields, Interstellar medium

## Abstract

Galaxies are observed to host magnetic fields with a typical total strength of around 15 $\upmu$G. A coherent large-scale field constitutes up to a few microgauss of the total, while the rest is built from strong magnetic fluctuations over a wide range of spatial scales. This represents sufficient magnetic energy for it to be dynamically significant. Several questions immediately arise: What is the physical mechanism that gives rise to such magnetic fields? How do these magnetic fields affect the formation and evolution of galaxies? In which physical processes do magnetic fields play a role, and how can that role be characterized? Numerical modelling of magnetized flows in galaxies is playing an ever-increasing role in finding those answers. We review major techniques used for these models. Current results strongly support the conclusion that field growth occurs during the formation of the first galaxies on timescales shorter than their accretion timescales due to small-scale turbulent dynamos. The saturated small-scale dynamo maintains field strengths at only a few percent of equipartition with turbulence. This is in contradiction with the observed magnitude of turbulent fields, but may be reconciled by the further contribution to the turbulent field of the large-scale dynamo. The subsequent action of large-scale dynamos in differentially rotating discs produces field strengths observed in low redshift galaxies, where it reaches equipartition with the turbulence and has substantial power at large scales. The field structure resulting appears consistent with observations including Faraday rotation and polarisation from synchrotron and dust thermal emission. Major remaining challenges include scaling numerical models toward realistic scale separations and Prandtl and Reynolds numbers.

## Introduction

Galaxies are observed to host magnetic fields with a typical total strength of around 15 $\upmu$G. A coherent large-scale field constitutes up to a few microgauss of the total, while the rest is built from strong magnetic fluctuations over a wide range of spatial scales (Beck 2015; Beck et al. 2019). Until recently, the importance of magnetic fields has often been overlooked when considering the dynamics and evolution of galaxies. However, magnetic field energy is sufficiently large to have a significance comparable to kinetic energy. Several questions immediately arise. What are the physical mechanisms that give rise to such strong magnetic fields? How do different physical processes in galaxies contribute to magnetic field growth? What are the small and large-scale structures of these fields? It is becoming increasingly clear that both small-scale dynamo (SSD) and large-scale dynamo (LSD) processes play a fundamental role in determining the answers to these questions. Progress in understanding dynamo behavior relies ever more strongly on numerical modelling of the magnetised plasma in galaxies. This review summarises the current state-of-the-art of these numerical simulations and their results. See also the recent review by Brandenburg and Ntormousi (2023).

### Fundamental observations of galactic magnetic fields

In galaxies, both the magnetic field and the velocity have structure across a wide spectrum of characteristic scales. First, there are contributions from the largest scales, such as the differential angular velocity that arises from the typical flat rotation curves with nearly constant rotation velocity as a function of radius observed in spiral galaxies embedded in dark matter halos. At slightly smaller scales, galactic outflows from galaxy centers or from powerful superbubbles produced by correlated SN explosions from young star clusters produce ordered velocities in the vertical direction outward from the galactic disc plane. Similarly, the magnetic field of spiral galaxies has been verified to have in most cases a coherent large-scale structure that sometimes, but by no means always, follows the optical spiral arms formed by the stars.

We have a variety of indirect observations through which we can identify the properties of the magnetic field in the interstellar medium (ISM), circumgalactic medium, intracluster medium (ICM) and, more locally, in molecular clouds, within and between spiral arms and around supernova (SN) remnants or superbubbles (see, e.g., Table 1 of Beck 2015). The total magnetic field $B_{\mathrm{tot_\perp }}$ perpendicular to the line of sight is measured through total synchrotron intensity. This is the sum of the ordered field $B_{\mathrm{{ord}}_\perp }$, which is defined as consistent within the beam width of the telescope, and the locally turbulent field $B_\mathrm{turb_\perp }$, which are the fluctuations on top of the regular field within the width of the beam, whether due to SSD, tangling or both. The regular field, $B_{\rm{reg}}$ which is considered the *mean field* within dynamo theory, is discriminated from the total field through polarised synchrotron emission. The sum of isotropic turbulent field $B_{\mathrm{iso_\perp }}$, with dispersions matching in all three dimensions, and anisotropic turbulent field $B_{\mathrm{aniso_\perp }}$, where dispersions impacted by compressions and shear differ, comprises $B_{\mathrm{turb_\perp }}$.

The isotropic turbulent field can be distinguished by combining unpolarised synchrotron intensity, beam depolarisation, and Faraday depolarisation (see, e.g., Ferrière 2020). The anistropic turbulent field can be difficult to distinguish from the regular field, so the ordered field energy $B_{\mathrm{ord_\perp }}^2=B_{\mathrm{reg_\perp }}^2+B_{\mathrm{aniso_\perp }}^2$. This is determined via intensity and radio, optical, infrared (IR), and submillimeter polarisation. The Goldreich and Kylafis (1981) and longitudinal Zeeman effects provide $B_{\mathrm{reg_\perp }}$ and $B_{\mathrm{reg_\parallel }}$, the latter being parallel to the line of sight.

**Fig. 1:**
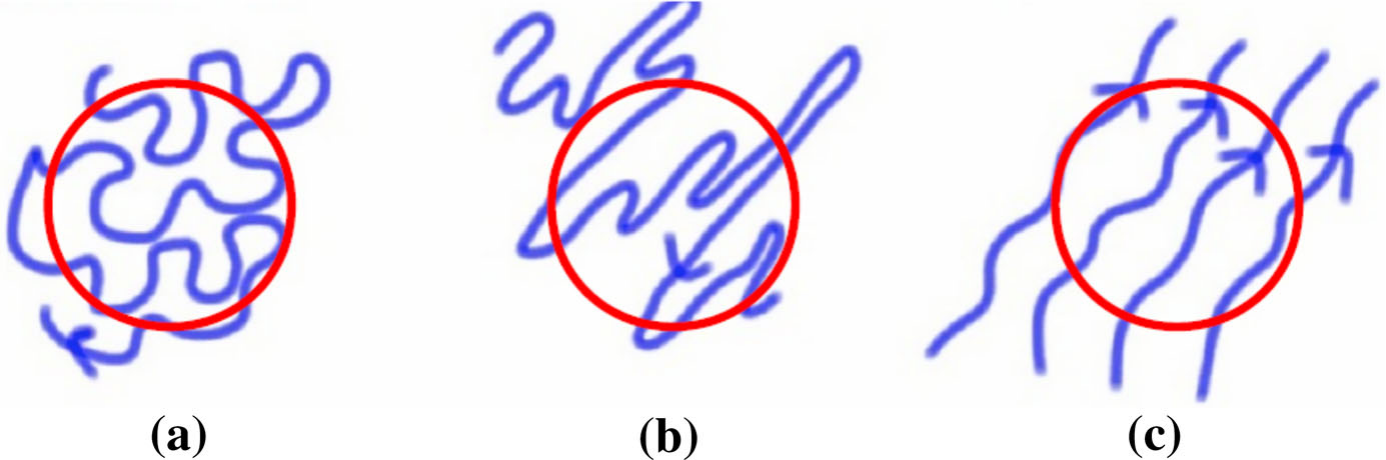
Schematic illustration of the composition of the magnetic field within a telescope beam broken down as **a** mostly $B_{\rm{iso}}$, **b** mostly $B_{\rm{aniso}}$ and **c** mostly $B_{\rm{reg}}$, with the red circle representing the telescope beam. Image reproduced with permission from Beck et al. (2019), courtesy Andrew Fletcher

To clarify this concept Fig. 1 illustrates how the isotropic random field $B_{\rm{iso}}$, the anisotropic random field $B_{\rm{aniso}}$, and the regular, or mean, field $B_{\rm{reg}}$ might be viewed. All three components will contribute to the total intensity of the unpolarised synchrotron emission. The ordered field is determined from the polarized emission within the width of the beam and includes the anisotropic random field, whose average within the beam is zero and the regular field, which has defined direction within the beam. The anisotropy, which can arise from preferential shearing of the random field by the localised flows, refers to the angle of the random field orientation, while its contribution to the mean field is zero, due to the reversals at scales smaller than the beam.

The polarisation angle would not distinguish the direction of the field, as in Fig. 1b, so cannot distinguish between the random and mean-field contribution. However, Faraday rotation is sensitive to the field direction and thus does discriminate the mean field. The distinction however depends on the width of the beam, so the higher the resolution the better the regular field can be traced. Current beam width resolution corresponds to a few hundred parsecs.

When interpreting observations, there is an underlying assumption that total magnetic field and total cosmic ray energy are in equipartition in regions emitting synchrotron radiation. There are various complications to deriving the field strength from the synchrotron intensity, which include the increased energy losses in cosmic rays (CRs), particularly electrons, in higher density environments, such as starburst regions and massive spiral arms, and the differences in the energy distribution of CRs produced by various sources, from which the integrated CR energy is obtained. Notwithstanding such uncertainties, a survey of 21 nearby galaxies obtained $B_{\rm{tot}}=17\pm 13\,\upmu \rm{G}$. Fletcher (2010) identify within this $B_{\rm{reg}} \simeq =5\pm 3\,\upmu \rm{G}$, but which has subsequently been more correctly relabelled $B_{\rm{ord}}$. A more reliable estimate of the regular field strength in spiral galaxies (Beck et al. 2019) is $1.7\pm 0.6\,\upmu \rm{G}$, because the ordered field is significantly comprised of anisotropic random fields. Fields as strong as 50–100 $\upmu$G are observed in starburst galaxies and some barred galaxies (Beck et al. 2005; Heesen et al. 2011; Adebahr et al. 2013). Such field strengths clearly indicate a magnetic field capable of exerting significant influences on the evolving dynamics and structure of the galaxies, including the processes leading to star formation. The relatively high strength of the turbulent field relative to the regular field is a common feature across observations on the scale of the Galaxy (see, e.g., Table 3 Beck et al. 2019), who determine $0.04\le B_{\rm{reg}}/B_{\rm{tot}}\le 0.43$ across 12 galaxies and at a variety of galactic radii. They observe a total field strength of only $13\pm 4\,\upmu \rm{G}$, although the Fletcher (2010) comparison includes non-spiral galaxies with exceptionally strong fields.

Even setting aside the uncertainties in the measurements of the magnetic field, there is no observational diagnostic beyond its overall magnitude to distinguish the turbulent field that arises through an SSD from that which results from tangling of the regular field by the turbulent flow. Recent high-resolution observations of extragalactic magnetic fields with *SOFIA* (Borlaff et al. 2021) identify polarisation fractions in the dense star forming regions traced by far-infrared (FIR) anticorrelated with the star formation rate, and generally lower than the polarisation of 3- and 6-cm radio emission. This is suggested to imply complex magnetised structures in the star-forming regions characteristic of SSDs acting there. An accurate model of the turbulent structure of the magnetic field in galaxies is required, not only to measure and understand the magnetic field, but also to correctly interpret its effect on how observables from distant sources reach the observer.

Tsouros et al. (2024) use a 3D map of observed dust density distribution in the local bubble of the Solar neighbourhood (Leike and Enßlin 2019; Leike et al. 2020) and simulated cosmic ray detection to infer the structure of the local magnetic field, assuming weak turbulence. Such observational inference provides a suitable benchmark against which to test numerical models. Insight derived from numerical modelling into the correlation between the field and velocities, gas and dust distributions will improve observational inference. From extragalactic sources, Brown et al. (2007) use RM to determine the mean magnetic field in the Milky Way. It aligns along spiral arms and has a strength up to about 2 $\upmu$G. Within alternating spiral arms it is observed to switch sign.

Such field reversals are not common in observations of external galaxies. Determinations of the magnetic field in M31 (Fletcher et al. 2004) and M51 (Fletcher et al. 2011) show the ordered and fluctuating fields to be of roughly equivalent energy, with strength 5 $\upmu$G each across a radial extent between 6 and 14 kpc. Non-axisymmetric configurations are certainly observed in the distribution of magnetic field (Ehle and Beck 1993; Chamandy et al. 2013a, b), as well as misalignment along the spiral arms (Fletcher et al. 2004, 2011; Fletcher 2010) and field reversals (Brown et al. 2007; Haverkorn 2015; Stein et al. 2019). In contradiction to Fletcher et al. (2004) findings of M31 pitch angles flattening with galactic radius, Mao et al. (2017) find pitch angles roughly constant at $-20^\circ$ from the azimuth. This may be explained by the pitch angles of the ordered anisotropic field not necessarily aligning with those of the regular field over distances exceeding the beam width.

At redshift $0.6< z <2$ galaxies have already been observed to have regular (mean) magnetic fields of order 10 $\upmu$G (Bernet et al. 2008). At redshift 0.4, Mao et al. (2017) obtain similar measurements. At redshift 2.6, they (see also Geach et al. 2023) show that much stronger ordered magnetic fields already existed, although unfortunately without any indication of the fraction of regular (dynamo-generated) field.

### Governing physical equations

Galactic dynamos are most often studied using the magnetohydrodynamics (MHD) framework, which describes the interactions of magnetic fields with a plasma that is treated as a continuum. The justification for these approximations rests on the relatively small mean-free path and the tiny Larmor radius of charged particles in the ISM

1 $$\begin{aligned} r_{\rm{L}} = \frac{mcv}{qB} = (36\, \rm{km}) \left( \frac{m}{m_{\rm{H}}}\right) \left( \frac{v}{10\, \mathrm {km\ s}^{-1}}\right) \left( \frac{{{\hat{e}}}}{q}\right) \left( \frac{3\, \upmu \rm{G}}{B}\right) \end{aligned}$$

where *m* and *q* are the mass and charge of the charged particles, *v* and *B* are the magnitudes of their perpendicular velocity and the ambient magnetic field, ${\rm{m}}_{\rm{H}} = 1.67 \times 10^{-24}\, \rm{g}$ and ${{\hat{e}}} = 4.8 \times 10^{-10}\, \rm{esu}$ are the mass and charge of a proton, and $c = 2.99 \times 10^{10}\, \mathrm {cm\ s}^{-1}$ is the speed of light.

Further, the plasma is usually resistive, that is, it has a physically significant magnetic diffusivity $\eta =\left( \mu _0 \sigma \right) ^{-1}\ne 0$, where $\mu _0$ is the vacuum permeability and $\sigma$ the electrical conductivity of the medium. A consequence of finite resistivity is that reconnection must occur at some rate. The details of reconnection have been argued to depend on kinetic effects (Yamada et al. 2010), but MHD turbulence may inevitably drive small-scale reconnecting structures regardless of kinetic scale effects (Eyink et al. 2011; Oishi et al. 2015; Lazarian et al. 2020). For modelling dynamo behavior the turbulent reconnection process can be usefully abstracted with Laplacian Ohmic resistivity.

The evolution of magnetic fields in an electrically conducting fluid is governed by the induction equation

2 $$\begin{aligned} \frac{\partial {{\varvec{B}}}}{\partial t}=\nabla \times \left( {{\varvec{u}}} \times {{\varvec{B}}} - \eta \nabla \times {{\varvec{B}}} \right) , \end{aligned}$$

where ${{\varvec{B}}}$ is the magnetic field and ${{\varvec{u}}}$ is the velocity field. The governing equation for the dynamics of viscous fluids is the Navier–Stokes equation, which can be written in the following non-conservative form for rotating, magnetised galaxies in a gravitational potential:

3 $$\begin{aligned} \rho \frac{D {{\varvec{u}}}}{Dt} = \rho {{\varvec{g}}} - {\varvec{\nabla }}p - 2{\rho } {\varvec{\Omega }} \times {\varvec{u}} {-\rho {\varvec{\Omega }} \times \left( {\varvec{\Omega }}\times {\varvec{x}}\right) } + {\varvec{J}} \times {\varvec{B}} + {\varvec{\nabla }} \cdot \left( 2 \rho \nu \textsf{S} \right) , \end{aligned}$$

where ${{\varvec{g}}}$ is the gravitational acceleration field, *p* is the thermal pressure, ${\varvec{\Omega }}$ is the rotation vector, ${\varvec{J}}$ is the current density, $\nu$ is the kinematic viscosity, and $\textsf{S}$ is the traceless rate of strain tensor, $\textsf{S}_{ij}= \frac{1}{2} \left( u_{i,j} + u_{j,i} \right) -\frac{1}{3} \delta _{ij} u_{k,k}$. Additional terms such as the cosmic ray pressure gradient, coupled to a model of cosmic ray production, destruction, advection, and energy loss and gain can also play a role. When the dynamical viscosity, $\rho \nu$, is homogeneous, then the viscous force can be simplified as

4 $$\begin{aligned} \nabla \cdot \left( 2 \rho \nu \textsf{S} \right) = \rho \nu \left( {\varvec{\nabla }}^2 {\varvec{u}} + \frac{1}{3} {\varvec{\nabla }} \left( {\varvec{\nabla }} \cdot {\varvec{u}} \right) \right) . \end{aligned}$$

The turbulent component of the velocity field can have its origin in the cascade of energy from large scales towards small scales thanks to the nonlinear interactions in the Navier–Stokes equation (Kolmogorov 1941). However in galaxies, deviating from the standard picture of forcing at largest scales driven, e.g., by large-scale shear, energy injection occurs at multiple scales from distributed stellar feedback in the form of ionising radiation, stellar winds, and SNe; gravitational instability of the gas disc; and, especially for young galaxies, gas accretion onto the galaxy (e.g., Klessen and Hennebelle 2010). The driving mechanisms for the turbulence also include stellar feedback. Ionising radiation contributes an order of magnitude more energy than the SNe (Abbott 1982), while SNe in turn contribute an order of magnitude more energy than stellar winds (Shull and Saken 1995). However, the momentum input from supernovae exceeds that from ionisation, as much of that energy goes to ionising and heating the gas. The resulting multiphase thermal structure of the ISM does have consequences for dynamos, as we will see in Sect. 3.3 below. The smallest contribution to driving has been estimated to originate from the large-scale shear, an order of magnitude less than the least powerful stellar source (Abbott 1982). Driving from stellar feedback causes mixing of the ISM at much smaller scales than those associated with systemic large-scale flows and ordered magnetic fields.

Conservation of mass implies

5 $$\begin{aligned} \frac{\partial \rho }{\partial t} + {\varvec{\nabla }}\cdot (\rho {\varvec{u}})=0, \end{aligned}$$

while conservation of thermal energy per unit mass *e* then implies

6 $$\begin{aligned} \rho \frac{De}{Dt} = -p{\varvec{\nabla }} \cdot {{\varvec{u}}} + {\varvec{\nabla }} \cdot {\varvec{q}} + 2 \rho \nu \vert \textsf{S}\vert ^2 + \eta \mu _0 {\varvec{J}}^2 + \rho \Gamma - \rho ^2\Lambda , \end{aligned}$$

where ${\varvec{q}}$ is the conductive heat flux and the last four terms describe the viscous and resistive heating and radiative heating and cooling. The radiative heating and cooling depend sensitively on the temperature, ionisation state, chemical abundances, metallicity, and dust properties (see Klessen and Glover 2016 for a comprehensive review of interstellar processes). The simplest approximation is to treat heating as a constant rate due to far-ultraviolet ejection of photoelectrons from dust and follow the cooling as a function of temperature. More complete models include descriptions of radiation transport and chemistry including both ionisation and molecule formation to determine both the heating and the cooling. The temperature-dependent structure of the cooling function allows gas in thermal equilibrium in an isobaric medium to exist at two temperatures for typical interstellar pressures, which are described as thermal phases (Field et al. 1969; Wolfire et al. 1995, 2003). Hot gas at low density takes sufficiently long to cool that it acts as a third quasistable phase (McKee and Ostriker 1977).

The most common way of closing the equations is to use the ideal gas law

7 $$\begin{aligned} p = (\gamma - 1) \rho e, \end{aligned}$$

where $\gamma =c_{\rm{P}}/c_{\rm{V}}$, $c_{\rm{P}}$ is the heat capacity at constant pressure, and $c_{\rm{V}}$ that at constant volume, providing the relation between density and pressure required to solve this set of equations. However, note that $\gamma$ also varies with the ionisation and chemical state of the gas, so in the general case this equation is position dependent.

In addition to the stellar feedback, the ISM can undergo instabilities of various kinds. Especially the magnetorotational instability (MRI), gravitational instability, and the instabilities caused by cosmic rays could be important for galactic dynamo action.

The MRI (Balbus and Hawley 1991; Piontek and Ostriker 2004, 2005, 2007) can alter both large- and small-scale dynamics. It has been semi-analytically argued, however, that this instability would be suppressed by intense SN activity up to a galactocentric radius of 15  kpc with Milky Way parameters (Korpi et al. 2010), but it could still affect the dynamics significantly in the outer regions (Sellwood and Balbus 1999) explaining the anomalously high velocity dispersions there (Tamburro et al. 2009; Beck 2015).

At smaller scales, SN remnant shells undergo fragmentation, e.g., due to the Richtmeyer–Meshkov instability (Rayleigh–Taylor instability due to acceleration rather than gravity) or either of the Vishniac instabilities (Vishniac 1983, 1994). These instabilities have been argued to be important in ’breaking up’ the shells of superbubbles and enable them to overcome the flux-freezing constraint and act favourably for a magneto-buoyantly driven dynamo (see, e.g., Kulsrud 2015). The theory for this instability, dubbed as the spike instability, has been developed in the early linear stage. It seems unlikely that the assumptions applied in this stage, such as treating the escape of magnetic flux as a diffusive process, are valid in the nonlinear stage, however.

Another way of triggering magneto-buoyancy in galactic disks is the Parker instability powered by cosmic rays released in SN explosions (Parker 1992). The significant pressure exterted by the cosmic rays enables the frozen-in magnetic field, even without fragmentation, to buoyantly rise up to the galactic halo. Meanwhile a portion of the gas will sink along the magnetic field lines of the so-formed loops, and finally form clumps at the connected footpoint of the flux tubes. These magnetic loops can be sheared by differential rotation, twist, and amplify the magnetic field (Hanasz and Lesch 1997).

Another instability induced by interstellar cosmic rays is the streaming instability. According to the analytic theory (Bell 2004), positively charged cosmic rays, streaming along a mean magnetic field, e.g., of LSD origin, induce constant currents along supernova-driven shock jumps in the density of the cosmic rays and background plasma. In the presence of a magnetic perturbation perpendicular to the shock, the action of the Lorentz force causes the background plasma to move transversely, while the cosmic ray plasma is much less affected. If the magnetic perturbations are helical, these motions can amplify the magnetic field by orders of magnitude (see, e.g., Riquelme and Spitkovsky 2009). Even though the scale of the plasma is much smaller than the Larmor radius of the background magnetic fields, this instability has recently been argued to cause significant effect on the scale of the small-scale magnetic field and interstellar turbulence (Riquelme and Spitkovsky 2009).

Gas-rich galactic disks characteristic of high-redshift galaxies have strong gravitational instabilities of the type described by Toomre (1964) and Goldreich and Lynden-Bell (1965). These drive turbulence from large scales independent of stellar feedback (e.g., Krumholz et al. 2018) that drive prompt SSD action early in galactic history (see Sect. 3.5 below). Furthermore, Klessen and Hennebelle (2010) showed that accretion flows, whether on to galaxies or individual star-forming cores, can drive substantial amounts of turbulence. These flows have been shown by numerical models to be dynamo active in the case of first star formation (e.g., Sur et al. 2010) and during galaxy formation (e.g., Pfrommer et al. 2022). For further review, see Sects. 3.2 and 3.4 below.

### Basics and challenges of dynamo theory

In order to model dynamos, both the velocity and magnetic field can naturally be decomposed into coherent and fluctuating parts, so that we can write ${{\varvec{u}}}=\overline{{{\varvec{u}}}}+ {{\varvec{u}}'}$ and ${{\varvec{B}}}=\overline{{{\varvec{B}}}}+ {{\varvec{b}}'}$, and follow their evolution separately. Ideally, the averaging should be over many independent ensembles of the turbulent velocity field, but in practice, spatial averages are used. One often uses Reynolds averaging obeying the following rules (Reynolds rules):

8 $$\begin{aligned} \overline{{\varvec{F}}_1 + {\varvec{F}}_2}= & {} \overline{{\varvec{F}}_1} + \overline{{\varvec{F}}_2}, \ \overline{\overline{{\varvec{F}}}} = \overline{{\varvec{F}}}, \ \overline{\overline{{\varvec{F}}{\varvec{f}}}} = 0, \ \overline{{\varvec{f}}}=0, \ \overline{\overline{{\varvec{F}}_1}\overline{{\varvec{F}}_2}} = \overline{{\varvec{F}}_1} \overline{{\varvec{F}}_2},\nonumber \\ \ \overline{\frac{\partial {\varvec{F}}}{\partial t}}= & {} \frac{\partial \overline{{\varvec{F}}}}{\partial t}, \ \overline{\frac{\partial {\varvec{F}}}{\partial x_i}} = \frac{\partial \overline{{\varvec{F}}}}{\partial x_i}, \end{aligned}$$

where ${\varvec{F}}_1$ and ${\varvec{F_2}}$ are mean fields and ${\varvec{f}}$ is a fluctuating field in a decomposition ${\varvec{F}} = \overline{{\varvec{F}}} + {\varvec{f}}$. If these rules hold, then no strict requirement for clear separation between the average and fluctuating scales exists, and the decomposition of the equations into these two parts is generally valid (see, e.g., Brandenburg and Subramanian 2005).

In the context of kiloparsec-scale Cartesian shearing-box models, see Sect. 4.1, *horizontal* averaging, i.e., averaging over the local radius and azimuthal extent of the computational domain, is often used when defining the mean fields and measuring the turbulent transport coefficients. This averaging obeys the Reynolds rules, but this choice can be criticised on at least two grounds. Firstly, many magnetic field configurations in galaxies are not axisymmetric, so a Fourier filtering based decomposition would be a more general choice for these. Secondly, observational diagnostics are hardly ever derived basing on such averaging. Gaussian filtering was successfully applied to decompose into the mean and fluctuating fields by Gent et al. (2013b), which would be a more consistent choice when comparing with observations (see, e.g., the discussion in Hollins et al. 2022). The major difficulty with these alternative averaging techniques is that neither of them obeys the Reynolds rules, although this is no hindrance to deriving alternative, mathematically sound formalisms for the mean quantities (e.g., Germano 1992).

#### Large-scale dynamo instability

Unless otherwise stated, in the following we will use generic Reynolds-rules obeying averages. After taking the average of the induction equation, we obtain an evolution equation for the mean field

9 $$\begin{aligned} \frac{\partial \overline{{{\varvec{B}}}}}{\partial t} = {\varvec{\nabla }} \times \left( \overline{{{\varvec{u}}}}\times \overline{{{\varvec{B}}}}+ {\varvec{\mathscr{E}}}- \eta {\varvec{\nabla }} \times \overline{{{\varvec{B}}}}\right) , \end{aligned}$$

where ${\varvec{\mathscr{E}}}= \overline{{{\varvec{u}}'}\times {{\varvec{b}}'}}$ is the turbulent electromotive force, which depends on the correlations of the velocity and magnetic fluctuations.^1^

The evolution equation for the magnetic fluctuations is obtained by subtracting the mean-field equation from the original induction equation:

10 $$\begin{aligned} \frac{\partial {{\varvec{b}}'}}{\partial t}={\varvec{\nabla }} \times \left( \overline{{{\varvec{u}}}}\times {{\varvec{b}}'}+ {{\varvec{u}}'}\times \overline{{{\varvec{B}}}}+ {{\varvec{u}}'}\times {{\varvec{b}}'}-{\varvec{\mathscr{E}}}-\eta {\varvec{\nabla }} \times {{\varvec{b}}'}\right) . \end{aligned}$$

Two regimes are usually distinguished, where the Lorentz force due to the magnetic field (1) is still weak, so that it will not back react on the flow, called the *kinematic regime*; (2) is dynamically significant, so the magnetic field influences the flow, called the *nonlinear regime*. In SSD-active systems also magnetic background fluctuations exist, ${{\varvec{b}}}_0^{'} \ne 0$, unrelated to the presence of $\overline{{{\varvec{B}}}}$, and the turbulent transport coefficients can depend on them (see, e.g., Rheinhardt and Brandenburg 2010).

In the kinematic regime, further restricting to the case with ${{\varvec{b}}}_0^{'}=0$, the presence of a mean magnetic field will induce a fluctuating magnetic field through the term ${{\varvec{u}}'}\times \overline{{{\varvec{B}}}}$. Therefore, even if ${{\varvec{u}}'}$ and ${{\varvec{b}}'}$ were uncorrelated without the mean magnetic field, in its presence they will be correlated so that ${\overline{{{\varvec{u}}'}\times {{\varvec{b}}'}}}$ is no longer zero, and in addition linearly correlated with $\overline{{{\varvec{B}}}}$. If $\overline{{{\varvec{B}}}}$ varies over scales much larger than those of the turbulent fluctuations, then one can expand ${\varvec{\mathscr{E}}}$ in a Taylor series expansion of the form

11 $$\begin{aligned} {\mathscr {E}}_i=a_{ij} {\overline{B}}_j+ b_{ijk} \frac{\partial {\overline{B}}_j}{\partial x_k}+\cdots \end{aligned}$$

and truncate after the first-order spatial derivatives of $\overline{{{\varvec{B}}}}$. Here, $a_{ij}$ and $b_{ijk}$ are tensors that describe the influence of turbulence on the evolution of the mean magnetic field, commonly described as turbulent transport coefficients. Particularly, the symmetric part of the ${\varvec{a}}$ tensor, usually denoted with ${\varvec{\alpha }}$, describes the collective inductive effect resulting from turbulent motions, and the symmetric part of the rank two tensor acting upon ${{\varvec{\nabla }}}\times \overline{{{\varvec{B}}}}$, usually denoted with ${\varvec{\beta }}$, describes the enhanced magnetic diffusivity resulting from them. In the kinematic regime, the transport coefficients may depend on the key hydrodynamic and thermodynamic quantities, but not on $\overline{{{\varvec{B}}}}$ itself. With this simplification, only the evolution of the magnetic fields at the largest scales can be captured. Simplifying even further, as done in the oldest and most traditional first-order smoothing approximation (FOSA) or second-order correlation approximation (SOCA) (see, e.g., Brandenburg and Subramanian 2005) the term ${\varvec{\nabla }} \times ({{\varvec{u}}'}\times {{\varvec{b}}'}- \overline{{{\varvec{u}}'}\times {{\varvec{b}}'}})$ is neglected in Eq. (10) for the fluctuating field. This is valid only when the magnetic Reynolds number, $\rm{Re}_{\rm{M}}\ll 1$, or when the Strouhal number

12 $$\begin{aligned} \hbox{St}=\frac{\tau u}{l}\ll 1, \end{aligned}$$

where $\tau$ is the correlation time of the turbulence, and *u* and *l* are typical velocity and length scales, respectively. Under one of these assumptions, the equation for the fluctuating field, Eq. (10), simplifies to the extent that it can be solved analytically for a given ${{\varvec{u}}'}$, after which ${\varvec{\mathscr{E}}}$ can be calculated with relative ease (Steenbeck et al. 1966).

Particularly, in the case of isotropic and homogeneous turbulence, the turbulent transport coefficients then reduce to scalars,

13 $$\begin{aligned} \alpha\approx & {} -\frac{1}{3} \tau \overline{{{\varvec{u}}'}\cdot \omega '}, \end{aligned}$$

14 $$\begin{aligned} \beta\approx & {} \frac{1}{3} \tau \overline{{{\varvec{u}}'}^2}, \end{aligned}$$

where ${\boldsymbol{\omega'}}$ is the fluctuating vorticity, and ${{\varvec{u}}'}\cdot {\boldsymbol{\omega'}}$ is the small-scale kinetic helicity of the turbulent flow. It can be seen that $\alpha$ relates to the kinetic helicity of turbulence, and $\beta$ to the intensity of the turbulent motions. A more generic form of the turbulent emf can be obtained by relaxing isotropy and homogeneity, which certainly can be appropriate in galaxies, where rotation and large-scale shear are present:

15 $$\begin{aligned} {\varvec{\mathscr{E}}}={{\varvec{\alpha }}}\cdot \overline{{{\varvec{B}}}}+{{\varvec{\gamma }}}\times \overline{{{\varvec{B}}}}-{{\varvec{\beta }}}\cdot ({\varvec{\nabla }} \times \overline{{{\varvec{B}}}}) -{{\varvec{\delta }}}\times ({\varvec{\nabla }} \times \overline{{{\varvec{B}}}}) -{{\varvec{\kappa }}} \cdot ({\varvec{\nabla }} \overline{{{\varvec{B}}}})^{(s)}, \end{aligned}$$

$\alpha$ and $\beta$ tensors now acquire off-diagonal elements. Completely new effects arise, as discussed, e.g., by Krause and Rädler (1980): ${\varvec{\gamma }}$ describes turbulent pumping of the mean magnetic field (e.g., Ossendrijver et al. 2002); ${\varvec{\delta }}$ encompasses new dynamo effects arising from current and rotation ($\Omega \times {\varvec{{\overline{J}}}}$ or Rädler effect Rädler 1969) or shear (shear-current effect Rogachevskii and Kleeorin 2004); $({\varvec{\nabla }} \overline{{{\varvec{B}}}})^{(s)}$ is the symmetric part of the derivative tensor of $\overline{{{\varvec{B}}}}$; and ${\varvec{\kappa }}$ is a rank-three tensor, which has also been shown to be important in the dynamo process by Warnecke et al. (2021), although it is neither fully diffusive nor inductive.

In the nonlinear regime, still restricting to the case ${{\varvec{b}}}_0^{'} = 0$, the turbulent transport coefficients may depend on both $\overline{{{\varvec{B}}}}$ and on ${{\varvec{b}}'}$. The outcome of taking into account these nonlinearities is strongly dependent on the closure adopted for Eq. (10) (see, e.g., Shukurov and Subramanian 2021). Until the turn of the millennium, most nonlinear galactic dynamo models adopted a heuristic closure, where mainly the effect of $\overline{{{\varvec{B}}}}$ on the $\alpha$ effect is postulated to be important, so that the inductive effect is suppressed when the mean field is approaching equipartition with the turbulence:

16 $$\begin{aligned} \alpha =\frac{\alpha _0}{1 + \vert \overline{{{\varvec{B}}}}\vert /\vert B_{\rm{eq}}\vert }. \end{aligned}$$

Here, $\alpha _0$ is the kinematic value of the $\alpha$ effect and $B_{\rm{eq}}\equiv \rho \overline{{{\varvec{u}}'}^2}$ is the field strength at equipartition with the turbulent kinetic energy. Evidence from numerical simulations in fully periodic domains in the beginning of the 1990s (e.g., Cattaneo and Vainshtein 1991; Vainshtein and Cattaneo 1992) forced the dynamo community to seriously consider other, more complicated and complete, closures, some of which had already been formulated years ago, (e.g., Pouquet et al. 1976; Vainshtein and Kichatinov 1983) and derive better nonlinear models based on them. Numerical simulations of forced turbulence, also exhibiting exponential amplification of magnetic fluctuations through the SSD (case of ${{\varvec{b}}}_0^{'} \ne 0$), showed that the $\alpha$ effect is not quenched according to Eq. (16), but in a catastrophic fashion, where also the magnetic Reynolds number appears in the denominator. Practically, this was interpreted to imply that galactic (or any astrophysical) $\alpha$-effect based dynamos should not exist.

However, this quenching behavior was later understood not to result from the SSD-generated fluctuations, but to be a consequence of magnetic helicity conservation (summarised e.g., by Brandenburg and Subramanian 2005): under the mean-field approximation, a dynamo produces helicities of equal magnitude but of opposite signs to satisfy helicity conservation. If no helicity fluxes escape the dynamo active region, then the small-scale magnetic helicity will quench the turbulent inductive $\alpha$ effect leading to the catastrophic quenching of the LSD. This is because the $\alpha$ effect acquires a magnetic modification, so that

17 $$\begin{aligned} \alpha \equiv \alpha _{\rm{K}} + \alpha _{\rm{m}}. \end{aligned}$$

One path into deriving the magnetic contribution to the $\alpha$ effect is the so-called minimal $\tau$ approximation, where the triple correlations appearing in the emf closure problem are modelled as a relaxation term (for details, see, e.g., Brandenburg and Subramanian 2005). The turbulent transport coefficients then read for incompressible flows

18 $$\begin{aligned} \alpha= & {} -\frac{\tau }{3} \left( \overline{{\varvec{\omega }}' \cdot {{\varvec{u}}'}} - \overline{{\varvec{j}}' \cdot {{\varvec{b}}'}} / {\overline{\rho }} \right) , \end{aligned}$$

19 $$\begin{aligned} \beta= & {} \frac{\tau }{3} \overline{{{\varvec{u}}'}^2}, \end{aligned}$$

where ${{\varvec{j}}}^{'} = {{\varvec{\nabla }}} \times {{\varvec{b}}}^{'}/\mu _{0}$ is the fluctuating current density, and ${\overline{\rho }}$ is the mean density. According to the magnetic helicity conservation law, the magnetic part has to satisfy (see, e.g., Brandenburg and Subramanian 2005) leading to an evolution equation

20 $$\begin{aligned} \frac{\partial \alpha _{{\rm m}}}{\partial t}= -\eta _{{\rm t}} k_0^2 \left( \frac{2 {\varvec{\mathscr{E}}}\cdot \overline{{{\varvec{B}}}}+ \nabla \cdot \overline{{{\varvec{F}}}}}{B_{{\rm eq}}^2} +\frac{2 \alpha _{{\rm m}}}{{\widetilde{{\rm Re}_{{\rm M}}}}}\right) . \end{aligned}$$

Here $\eta _{\rm{t}} = \eta +\beta$ is the sum of microscopic resistivity, $\eta$, and the enhanced turbulent resistivity, $\beta$, $\overline{{\varvec{F}}}$ is the large-scale helicity flux of the fluctuating field, $k_0$ is the wavenumber corresponding to the integral scale of turbulence, and ${\widetilde{\rm{Re}_{\rm{M}}}} \equiv \eta _{\rm{t}}/\eta$. The most advanced mean-field (MF) models for galaxies solve this equation together with the mean-field induction equation. With some plausible estimates from observations or theory for the coefficients of the models, galactic MF dynamos remain useful tools (see, e.g., Shukurov and Subramanian 2021), but verifying their applicability rigorously remains an open question (see the discussion in Sect. 3.4.1).

#### Small-scale dynamo instability

**Fig. 2:**
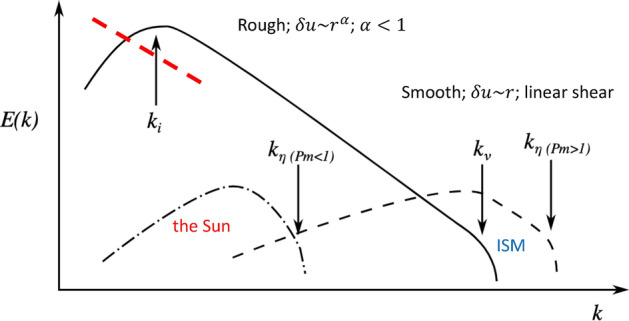
Schematic illustration of different types of SSD spectra. The solid line represents the kinetic energy spectrum, with a cut-off scale $k_{\nu }$, $\nu$ being the kinematic viscosity. The dashed lines represent magnetic energy spectra in the kinematic stage, with cut-off scales $k_{\eta }$, $\eta$ being the magnetic resistivity. Two different scenarios, differentiated by the magnetic Prandtl number, $\rm{P}_{\rm{m}}$ (in our notation $\rm{Pr}_{\rm{M}})=\nu /\eta$, are shown, corresponding for low (such as the Sun) and high (such as the ISM). The red long-dashed line illustrates the contribution of a LSD at the low wavenumber range. Image adapted from Rempel et al. (2023)

The ISM of galaxies is, in general, a high magnetic Prandtl number fluid, meaning that the ratio of magnetic to fluid Reynolds numbers

21 $$\begin{aligned} \rm{Pr}_{\rm{M}}\equiv \frac{\nu }{\eta }=\frac{\rm{Re}_{\rm{M}}}{\rm{Re}}, \end{aligned}$$

is large; Brandenburg and Subramanian (2005) estimate values of $\rm{Pr}_{\rm{M}}\sim {\mathscr {O}}(10^{11})$. The resistivity, $\eta$, is then much smaller than the fluid viscosity, $\nu$, meaning that the dissipation scale of the fluid motions far exceeds that of the magnetic field. This implies that the magnetic fields can dissipate into much smaller-scale structures than the fluid motions themselves, and hence a typical magnetic field structure sees the velocity field as a smooth, larger-scale shearing flow acting on it (compare spectra plotted with solid and dashed lines plotted in Fig. 2).

In such a setting, a rapid amplification of magnetic fluctuations by the smooth fluid motions can occur, independent of the flow possessing helicity or not. This dynamo instability is called the SSD or fluctuation dynamo. The scenario was first discussed by Batchelor (1950), and the first rigorous treatment of the non-helical case was presented in Kazantsev (1968). The amplification occurs at the timescale of the turbulent eddy turnover time, which is far shorter than the timescale required for the amplification of the large-scale field by an LSD. In the kinematic growth phase, the structures generated have a thickness around the resistive scale, but are curved up to the scale of the turbulent eddies. The only requirement for this instability to set in is that $\rm{Re}_{\rm{M}}$ must exceed a critical value, analytically derived to be 30–60, a condition that is fulfilled from the integral to the dissipation scale in galaxies (see e.g., Subramanian 1998).

Presumably, the setting in which the SSD starts to operate in galaxies is one where the LSD has not yet produced a significant large-scale field, but battery mechanisms and plasma instabilities can produce seed fields at small scales in the regime of dissipation scales rather than within the inertial range (see Sects. 1.4 and 3.1). Therefore, the SSD excitation likely starts in a diffusion-dominated setting, in contrast to the other possible, but unlikely, scenario of SSD starting with larger-scale tangled fields, in the diffusion-free regime (Rincon 2019). In the kinematic stage, when the magnetic fluctuations are still weak, the peak in the magnetic power spectrum is at the resistive scale, but all scales grow with the same growth rate. At scales larger than the resistive scale, the magnetic power spectrum is predicted to follow a positive power law of $k^{3/2}$ (Kazantsev 1968), called the Kazantsev spectrum. At smaller scales, the spectrum falls off steeply, following the Macdonald function (for details, see e.g., Brandenburg and Subramanian 2005; Rincon 2019). For a schematic representation of the magnetic spectrum, see Fig. 2 dashed line.

How the SSD nonlinearly saturates remains an open problem. By extending the Kazantsev model, it has been proposed that the SSD grows magnetic fluctuations at the resistive scale up to and exceeding equipartition with the kinetic energy of the turbulence, but that the generated field would be concentrated into resistive-scale ropes, hence not being volume filling, and therefore being energetically insignificant (e.g., Subramanian 1998). Whether reconnection plays a role in the nonlinear regime of SSD is a relatively fresh research question arising from such numerical research that is discussed in this review.

In galaxies that have already excited LSD, another source of magnetic fluctuations comes through tangling of the coherent component by the action of the turbulent eddies. Currently, the fraction of fluctuations caused by the SSD versus tangling is not known observationally, and calls for numerical approaches. Ideally, models where both SSD and LSD instabilities and their nonlinear interactions and saturation regimes are realistically captured are required to study the relation of the regular and fluctuating components. Such modelling requires large scale separation ratios, and thereby high resolution, and the models need to be integrated over timescales of gigayears. Currently, numerical models reach only modest magnetic Prandtl number regimes, while extending to the extreme regimes in galaxies is yet impossible and likely to remain that way in the near future. Even when working in parameter regimes far-removed from the ISM, numerical simulations with modest Prandtl numbers have provided some answers, and here we review the current state-of-the-art.

### Seed fields

Both the SSD and the LSD need initial magnetic fields to grow, as their evolution equations are homogeneous in $\overline{{{\varvec{B}}}}$ and ${{\varvec{b}}'}$, respectively. There have been a number of mechanisms proposed to generate such seeds. These have included seeds of cosmological origin (see, e.g., the review by Durrer and Neronov 2013); battery mechanisms (see, e.g., Subramanian et al. 1994; Durrive and Langer 2015); and stars picking up the battery fields during star formation, amplifying them through a stellar dynamo, and then ejecting the fields as seeds in SN explosions (Ruzmaikin et al. 1988; Martin-Alvarez et al. 2021).

The Biermann battery, which operates through the baroclinic effect when density and temperature gradients are misaligned, has been proposed to lead to seed fields organized on scales of cosmic ionization fronts (Subramanian et al. 1994; Kulsrud et al. 1997; Gnedin et al. 2000). The Durrive battery (Durrive and Langer 2015), in addition to the Biermann battery effect, assumes that an excess of momentum is gained by an electron after an atom is ionized, creating another contribution to the electromotive force. During the epoch of re-ionization, after the birth of the first massive stars, such ionized regions were created around them due to their radiation pressure. Durrive and Langer (2015) estimate that this mechanism leads to the existence of hundreds of parsec to kiloparsec scale seed fields of maximally $10^{-19}$G.

The generation of magnetic fields by cosmic accretion shocks from unmagnetised plasma was hypothesised by Gruzinov (2001) based on consideration of the properties of gamma ray burst shocks. Schlickeiser and Shukla (2003) and Lazar et al. (2009) made analytic arguments for the Weibel (1959) instability in shocks being the physical mechanism that could generate these fields when the shocks drive streams of electrons and ions moving in different directions. These ideas were supported by initial models of homogeneous Weibel instability performed with particle-in-cell codes by Medvedev et al. (2004) and Sakai et al. (2004). These fields are initially generated at small characteristic length scales, of the order of the electron skin depth. In Sect. 3.1 we discuss how a SSD has been demonstrated to both increase their length scales and amplify their magnitudes, potentially to astrophysically interesting values.

## Numerical approaches

### Mean-field modelling

The simplest numerical models use the approximations discussed in Sect. 1.3.1 to study the evolution of the large-scale component of the galactic magnetic field. Furthermore, as the rotation laws of galaxies are well-constrained observationally, the models most often work in the limit where the large-scale velocity field is steady instead of being evolved according to the mean-field Navier–Stokes equation. As such dynamo models do not solve for the full dynamical problem, but only for the evolution of the mean magnetic field (Eq. 9), they need ad hoc parameterisations of the turbulent transport coefficients. A comprehensive review of the MF approach and the results with given differential rotation profiles has recently been presented in Shukurov and Subramanian (2021). We summarise their theoretical discussion in the following, while for details and the results in the limit of given differential rotation laws we refer to their Chapters 11 and 12.

The dominant turbulent effects in galaxies are assumed to be the $\alpha$ effect and the enhanced turbulent diffusion, while turbulent pumping and the more exotic dynamo effects discussed in Sect. 1.3.1, are usually regarded to be sub-dominant. Furthermore, the scalar expressions for isotropic and homogeneous turbulence, Eqs. (13) and (14), are often assumed to be valid. As discussed earlier, it is observationally well-established that strong magnetic fluctuations dominate over the mean component in galaxies. The effect of the fluctuations, most likely implying strong pre-existing magnetic background turbulence ($b_{0}^{'} \ne$ 0), are currently not taken into account through the turbulent transport coefficients as such, but rather through their nonlinear saturation formulae, Eqs. (17)–(20). We note, however, that work into the direction of examining the explicit dependencies of the turbulent transport coefficients on the magnetic background turbulence is vividly ongoing, see (e.g., Chamandy and Singh 2017, 2018).

Observations allow for estimates of the magnitudes of $\alpha$ and $\beta$, and also that of the amplifying effect of differential rotation, making galactic dynamos better constrained than many other astrophysical dynamos, such as the solar one. Nevertheless, confirmations from numerical models, not working under the MF model assumptions, are required to test the validity of the various assumptions used, and to fill in details that the observations can not reveal due to the limited accuracy and projection effects. Such attempts, tools used, and the results obtained are discussed in Sect. 4.1.

Cylindrical coordinates ($r,\phi ,z$), with the *z*-axis aligned with the rotation vector, and $(r,\phi )$ being coordinates in the horizontal plane, are the most often used coordinates to simulate the MF equation. Under the assumption of axisymmetry, $\partial /\partial \phi$ vanishes. If we can further assume that the disc is very thin, that is, $\epsilon =h_0/R \ll 1$, where $h_0$ is the scale height and *R* is the galactocentric radius, one may also neglect all the terms with radial derivatives, so the evolution equation for $B_z$ decouples from that of the horizontal components. One is then left with the problem of solving the evolution equations for the radial and azimuthal components. In this approximation, the MF equations contain only *z*-derivatives, but the turbulent transport coefficients and the mean field itself can depend parametrically on the radius, and are thus local. The non-dimensional MF equations contain two control parameters for the LSD, called dynamo numbers:

22 $$\begin{aligned} R_{\Omega }=\frac{S_0 h_0^2}{\beta }, {\ \ } R_{\alpha }=\frac{\alpha _0 h_0}{\beta }, \end{aligned}$$

where ‘0’ subscripts now refer to characteristic values determined, e.g., from observations, and *S* describes the shear, for flat galactic rotation curves $S=-V_0/R$ being a good approximation, with $V_0$ being the roughly constant rotational velocity. Their product $D=R_{\alpha } R_{\Omega }$ characterises the overall dynamo efficiency. If we adopt the so-called $\alpha \Omega$ assumption, where the azimuthal magnetic field is solely generated by differential rotation, neglecting a term arising from the $\alpha$ effect, that can be estimated to be much smaller, then the parameter *D* is the only non-dimensional control parameter describing the full problem. Using qualitative arguments, that is, not solving any dynamo equations yet, the growth rate $\lambda$ is expected to scale as

23 $$\begin{aligned} \lambda ^{-1} \approx \frac{h_0^2}{\beta }| D |^{-1/2}. \end{aligned}$$

For numerical solutions, vacuum boundary conditions are often used, the justification coming from the estimate that the magnetic diffusivity beyond the disc is one to two orders of magnitude higher than in it, and the large-scale magnetic field is largely confined to the thin disc.

The assumption of axisymmetry is a strong restriction, as many galaxies have been observed to host non-axisymmetric magnetic field configurations (Sect. 1.1). The approach outlined above can, however, be relatively easily applied to a non-axisymmetric system as well. Now the parametric dependence of the mean magnetic field not only includes *R*, but also the azimuthal angle $\phi$. The turbulent transport coefficients can be either axi- or non-axisymmetric. There is one major difference to be accounted for: differential rotation acts differently on axi- versus non-axisymmetric solutions, efficiently destroying the latter, while the $\alpha$ effect does not make such a strong distinction. Hence, the $\alpha \Omega$ approximation is not applicable in this regime, and both dynamo numbers must be kept in the problem. Again, for very thin discs, an asymptotic solution can be constructed, where radial derivatives vanish, and the evolution equation for the *z*-component decouples. Qualitative considerations show that the main characteristics of the dynamo solutions, such as the growth rate, should remain invariant with respect to the axisymmetric solutions, implying a difficulty in explaining the observed dominantly non-axisymmetric magnetic fields in some galaxies with such simple models. Non-axisymmetric solutions have found to become possible only when non-axisymmetric disk properties, such as non-axisymmetric mean flows due to spiral arms or a bar or modulation of the turbulent transport coefficients have been considered (see, e.g., Chamandy et al. 2013a).

The fundamental process through which galactic dynamos are expected to saturate is magnetic helicity conservation, leading to the dynamical $\alpha$ effect, Eq. (20). Galactic discs, although often treated in MF models as thin discs, interact strongly with their environments, with stellar feedback driving galactic fountains and winds and material accreting from the surrounding circumgalactic medium (e.g., Tumlinson et al. 2017). Under such conditions, magnetic field is expected to be transported outwards when tied to the hot, ionised gas in fountains and winds, with some of it falling back with cooler and denser structures in the fountains and during accretion. Also, a turbulent pumping effect may be important here in transporting mean field inwards or outwards. All these processes will result in re-distribution of magnetic helicity at different scales, and helicity fluxes between different regions. Parameterising these effects in terms of helicity fluxes from the existing numerical models is a tremendous challenge. What is currently assumed in the MF models is described in Sect. 3.4.1, and what is known from numerical simulations is discussed in Sect. 4.2.

### Direct numerical simulation versus large eddy schemes

Nonlinear numerical simulation of dynamo action requires that resistivity be added, either explicitly or by discretisation error, to the equations of ideal MHD. The discretisation error present in any numerical method does provide a nonlinear resistivity that can enable or even suppress dynamo activity. Theoretically, an LSD can be excited whenever $\rm{Re}_{\rm{M}}>1$, while an SSD is known to require higher threshold values of order of tens to hundreds (see Sects. 1.3.1 and 1.3.2). Therefore, an LSD should be easier to model numerically than an SSD, but an LSD requires many more pre-requisites from turbulence, such as the standard expectation of small-scale flows being helical, to be satisfied than an SSD.

Including explicit diffusivities in numerical models at the extremely small values characteristic of the ISM, while retaining their correct ratio as reflected by the extremely large $\rm{Pr}_{\rm{M}}$ in the ISM, is currently an unreachable challenge. Often models including explicit diffusivity are called direct numerical simulations (DNS), but this denotion is not strictly valid due to the orders of magnitude elevated diffusivities required to exceed numerical diffusivity. A more proper term of DNS-like models has recently been proposed (Rempel et al. 2023). To be useful, such implementations must be run at high enough resolution that the numerical resistivity does not dominate over the physical values chosen. A parameter study to find the resolution where the physical resistivity starts to determine the solution must be done to demonstrate that numerical resistivity is not dominant.

Using explicit diffusivities has the disadvantage that it provides constant diffusion everywhere, while the necessity for using diffusion is the largest at the grid scale to ensure numerical stability and allow magnetic field lines to reconnect, while no such need exists for the resolved scales. Hence, implicit large-eddy simulations (ILES) are often used, where no explicit physical diffusion terms are included, but numerical diffusion remains active. In astrophysical modeling, the most usual sources of numerical diffusion are provided by truncation error in Riemann solvers (e.g., Balsara et al. 2004) or hyperdiffusivities introduced for numerical stability (Brandenburg and Dobler 2002; Pencil Code Collaboration et al. 2021). Grete et al. (2023) recently showed that such ILES schemes produce results that compare surprisingly well to the results of DNS-like models with well-resolved dissipation scales. The disadvantage of these sorts of ILES schemes is that control parameters such as $\rm{Re}$, $\rm{Re}_{\rm{M}}$, and $\rm{Pr}_{\rm{M}}$ become ill-defined. To counter-act this shortcoming, measurements have been performed of numerical resistivity (Rembiasz et al. 2017; McKee et al. 2020; Bian et al. 2021) and of numerical viscosity (Schranner et al. 2015; Obergaulinger and Aloy 2020; McKee et al. 2020; Bian et al. 2021) by comparing results of models without physical diffusivity to analytical or numerical solutions including physical diffusivity. Despite these efforts, the ILES approach remains unsatisfactory, as the diffusivity has a steep, unphysical scale dependence, with all diffusivity focused at scales close to the grid.

Explicit large-eddy simulations (ELES), where the solved equations are filtered at an intermediate scale, and the smaller-scale effects are described through a (ideally) physically motivated sub-grid-scale (SGS) model, have been developed for idealized MHD turbulence (Smagorinsky 1963; Grete et al. 2017). Although SGS schemes to model processes as SNe and star formation are popular, numerical SGS models attempting to describe the dynamo processes in the ISM remain scarce. One recent example is the model by Liu et al. (2022) that includes an unresolved turbulent dynamo. The most complicated case for the ELES schemes is presented by an LSD, in which small-scale, unresolved, effects on the large-scale dynamics, usually referred to as back-scatter in the LES framework, should be captured by the SGS model. Steps in the direction of a true ELES MHD model for supersonic turbulence have been taken by Grete et al. (2015) and Grete et al. (2017), but such an SGS model has not yet been incorporated into galactic dynamo simulations.

### Spectral methods

The first published numerical simulation of a turbulent dynamo was performed with a spectral method (Meneguzzi et al. 1981). Spectral methods have the highest accuracy derivative per grid point in smooth flows (e.g., Maron et al. 2008), but struggle to simulate the super-Alfvenic flows characteristic of the interstellar medium because of Gibbs ringing at shocks, so they have not featured prominently in studies of galactic dynamos.

### Structured grid methods

#### Uniform grids

Discretisation of the MHD equations onto a uniform Cartesian grid provides the most straightforward numerical approach. For the dynamo problem, a prime advantage of uniform grid methods available in codes such as the Pencil Code (Brandenburg and Dobler 2002; Pencil Code Collaboration et al. 2021), Athena (Stone et al. 2008), Athena++ (Stone et al. 2020), ZEUS-MP (Hayes et al. 2006), NIRVANA-III (Ziegler 2004, 2011), or Piernik (Hanasz et al. 2010a, b) is the uniform numerical dissipation properties across the computational domain. This advantage is unfortunately lost in the non-Cartesian geometries available in these codes, where different grid zones have different sizes or aspect ratios. Kritsuk et al. (2011) compares single-grid algorithms for isothermal, magnetised, supersonic turbulence showing that algorithmic numerical diffusivity and resolution can be traded against each other to get equally good solutions.

#### Hierarchical grids

Adaptive mesh refinement (AMR) methods focus resolution in subgrids into regions of interest, typically where strong gradients occur. Kritsuk et al. (2006) demonstrated in isothermal, supersonic, hydrodynamic turbulence without an explicit viscosity using Enzo (Collins et al. 2011) that if the driving and dissipation scales are well separated, and the base grid has sufficient resolution to resolve the larger scales of the turbulent cascade, then AMR of dissipation regions can reduce the cost of the simulation without changing the statistical properties of the turbulence. Li et al. (2012) similarly demonstrated using the ORION2 AMR MHD code that the statistics of magnetised turbulence can equally well be modeled with AMR methods, though with an emphasis on the properties of the highest density, highest resolution regions at the cost of the low density regions.

AMR methods have been applied to SSDs in dwarf galaxies by Rieder and Teyssier (2016) and Rieder and Teyssier (2017), and placed in a cosmological context in Rieder and Teyssier (2017). Martin-Alvarez et al. (2022) argue that to study SSDs in cosmological simulations of galaxies, refinement down to a uniform grid encompassing the galactic disc yields better results than a refinement strategy strictly tied to density gradients that results in lower resolution in low-density warm regions.

### Unstructured methods

There are multiple methods used for MHD dynamo simulations that use particles as fluid sampling points, either in a strictly Lagrangian treatment, or as generating points for a quasi-Lagrangian unstructured mesh. These include smoothed particle hydrodynamics (SPH), meshless finite mesh (MFM) and meshless finite volume (MFV) methods, and Riemann solvers on a Voronoi mesh. All of these methods generally have spatially varying resolution and thus spatially varying numerical diffusivity. In particular, Lagrangian or quasi-Lagrangian methods have higher resolution in dense regions than in rarefied regions. However, dynamo action can be strongest in hot, rarefied regions (see Sects. 3.3.1 and 3.4.2) that are poorly resolved and thus have SSD suppressed numerically when modeled with these methods.

Dolag and Stasyszyn (2009) implements MHD in the GADGET SPH code (Springel 2005) using a Powell et al. (1999) eight-wave divergence cleaning algorithm, which diffuses magnetic divergence across the grid to constrain it at a low level. This was used by subsequent publications for study of galactic field evolution in isolation or cosmological context (e.g., Beck et al. 2012, 2013; Steinwandel et al. 2019, 2020). An Euler potential approach (Price and Bate 2007) is implemented by Kotarba et al. (2009) into the VINE SPH code (Wetzstein et al. 2009), providing excellent conservation of field divergence but constraining magnetic helicity unphysically. Wissing and Shen (2020) implements MHD within the geometric density averaged force algorithm used by the Gasoline2 SPH code (Wadsley et al. 2017). This was used by Wissing and Shen (2023) to model dynamo activity in an isolated disc galaxy. However, these methods remain problematic because of the lack of zero-order convergence (Morris 1996; Dilts 1999; Read et al. 2010) and the large kernel sizes needed to run modern versions stably.

Pakmor and Springel (2013) describes the implementation of MHD in the Voronoi mesh code AREPO (Springel 2010) using an approximate Riemann solver to derive fluxes across the mesh edges. They used a Powell et al. (1999) scheme to maintain field divergence small. This was used by Pakmor et al. (2014) and Pakmor et al. (2017) to simulate galactic dynamos in cosmological context. Mocz et al. (2016) showed how to use constrained transport (Evans and Hawley 1988) with AREPO, to maintain zero divergence to machine precision even on the unstructured mesh.

An alternative approach to modelling fluid flow with particles is offered by weighted meshless schemes (Gaburov and Nitadori 2011), as implemented in the MFV and MFM methods used in GIZMO (Hopkins 2015). These methods use values at a particle and its neighbors to fit a polynomial to the solution and its derivatives, rather than averaging over the values and then taking derivatives using differences of the averages as SPH does. The difference between the two GIZMO methods lies in the decision of whether the Riemann problem at zone faces should be solved in the frame of motion of the fluid at the face (MFM) or at the mean cell velocity (MFV). MHD is implemented with a Godunov HLLD solver and a Dedner et al. (2002) magnetic divergence damping scheme.

### Hybrid and kinetic methods

Dynamos in collisionless plasmas, such as those found in the intracluster medium surrounding galaxies or in the hot early universe, can not be properly modeled with the MHD approximation because of the presence of fast kinetic instabilities. Rincon et al. (2016) used a hybrid model that treats protons as kinetic particles and electrons as an isothermal fluid to study this problem. Pusztai et al. (2020) used a fully kinetic model implemented with a discrete Galerkin solution of the kinetic Boltzmann equation limited to subviscous scales for computational tractability. Sironi et al. (2023) and Zhou et al. (2024) describe fluctuation dynamos modeled with a particle-in-cell treatment of an electron–positron pair plasma. These methods are vital for understanding the origin of seed fields, but are less relevant for most flows within the bulk ISM of galaxies. Extending them to include electron-ion mass ratios and greater dynamic range in scale will be essential for making contact with the physical parameter ranges occurring during galaxy formation.

## Results from numerical approaches

Simulations of dynamo action in galaxies have demonstrated its importance at all scales. Many expectations from analytical theory are supported, and the detailed field structure and evolution can be traced in this highly nonlinear problem. However, limitations of numerical resolution and the different numerical approximations used can result in qualitatively different outcomes in various situations, necessitating careful evaluation of reported results.

### Plasma scale

SSD action itself, working on the seed fields provided by either battery mechanisms or the Weibel (1959) instability (see Sect. 1.4) is a rapid amplifier of those weak fields, acting to provide a strong enough field for LSD to amplify the largest scale fields in galaxies to observed values (see, e.g., Beck et al. 1994; Bhat et al. 2016).

Rincon et al. (2016) demonstrated that magnetic field formation from an unmagnetised turbulent plasma and subsequent dynamo growth can occur. They used a hybrid model that includes an electron fluid and proton particles computed using a full six-dimensional Vlasov equation. This model suppresses both the Biermann (1950) battery and Weibel (1959) instability because of its assumption of isothermal electrons. However, Pusztai et al. (2020) used their fully kinetic model to demonstrate that Landau damping can limit the growth of kinetic dynamos in some regimes, an effect that cannot be seen in the hybrid approximation.

**Fig. 3:**
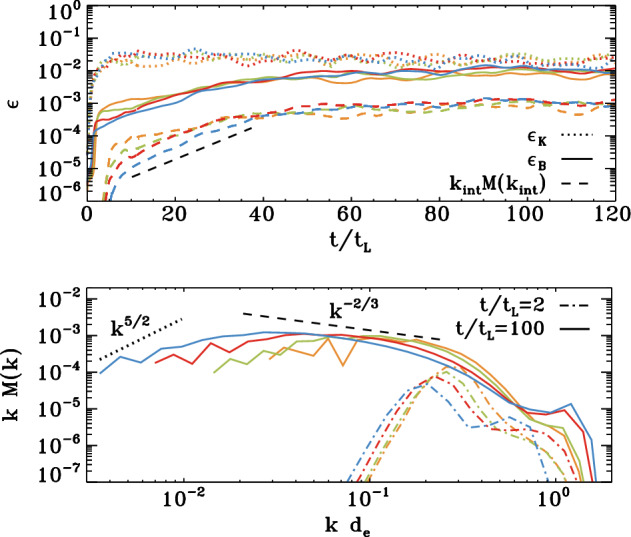
Kinetic models of an initially unmagnetised turbulent flow. **Top** Magnetic energy $\epsilon _B$ *(solid lines)*, kinetic energy $\epsilon _K$ *(dotted lines)*, and magnetic energy at the turbulent driving scale $k_{\rm{int}}$. Time is given in units of the crossing time $t_L$ at $k_{\rm{int}}$ for models with domain sizes *L* in terms of the electron skin depth $d_e$ of $L/d_e$ ranging from 250 *(orange)* to 2000 *(blue)*. The dashed black line shows exponential growth of the rms field $B_{\rm{rms}} \propto \exp (0.4v_{\rm{rms}} t / L)$. **Bottom** Magnetic power spectra with wavenumber *k* normalized by $d_e$ immediately after magnetisation by Weibel instability *(dot-dashed lines)* and in the quasisteady state after dynamo saturation at equipartition *(solid lines)*, with the Kazantsev ($M(k) \propto k^{3/2}$ and Kolmogorov $(M(k) \propto k^{-5/3}$ spectra shown in *black*. Note the decrease in the wavenumber of peak power as the domain size increases. Image reproduced with permission Sironi et al. (2023), copyright APS

Sironi et al. (2023) and Zhou et al. (2024) show that magnetic fields generated by the Weibel (1959) instability from an unmagnetised plasma can grow by SSD in driven turbulence, as shown in Fig. 3. These models include both positively and negatively charged particles in a fully kinetic simulation of a hot pair plasma, which excludes the Biermann (1950) battery effect. The SSD amplifies the magnetic energy to within a few percent of equipartition with the driving turbulence. It also increases the characteristic length scale to be close to the driving scale, as shown in the figure by the comparison of the power spectrum for increasing domain sizes. As the domain size and the driving scale $k_{\rm{int}}$ increase, the peak power moves to correspondingly smaller wave number. This mechanism might well be able to be extrapolated to astrophysically interesting length scales.

The separation between driving and dissipation scales in these kinetic models remains far smaller than in the astrophysical cases of galactic or intergalactic gas, and models still need to be done with more appropriate ratios of electron to ion masses. Nevertheless, the mechanisms described seem robust enough that we should conclude that such an SSD could occur already behind the accretion shocks that occur during the assembly of gas into galaxies. As a result, magnetic fields of at least a few percent of equipartition with turbulence likely provide the initial condition for *any* galactic dynamo. Simulations starting with extremely small seed fields may unphysically delay the onset of magnetohydrodynamic effects in galactic evolution.

### Star formation scale

During the formation of the first stars, dynamo action can already occur in the turbulent accretion flows assembling the gas into haloes. Kinetic simulations of this process were discussed in the previous section. Schleicher et al. (2010) presented analytic estimates of the resulting field strengths in halos, showing that near equipartition fields could already be expected even prior to the formation of the first stars.

After the accreted gas cools sufficiently to produce an accretion disc surrounding a protostar, magnetorotational instability in this disc can drive an SSD (Brandenburg et al. 1995). A semi-analytic model of the SSD in such a disc by Schober et al. (2012) established that fast growth would be expected. This treatment was extended to quickly accreting young galaxies by Schober et al. (2013).

**Fig. 4:**
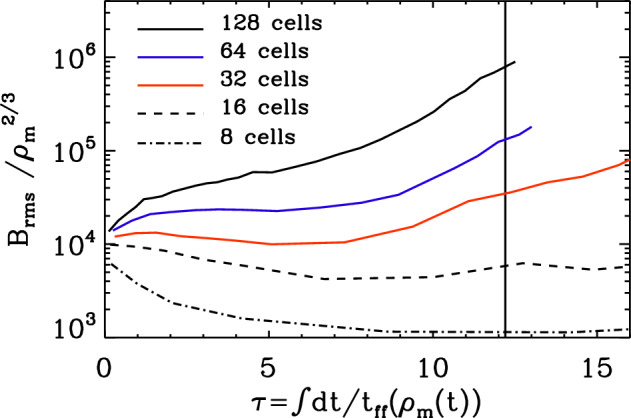
The number of cells with which the Jeans length is resolved in a primordial star formation region determines whether growth of the magnetic field from an SSD can be resolved. Amplification by spherical gravitational collapse produces field growth proportional to $\rho ^{2/3}$, so further field growth is evidence of the action of the SSD. Image reproduced with permission from Sur et al. (2010), copyright AAS

After the action of an SSD during accretion treated in the previous section, dynamos in protostellar accretion discs are another source of macroscopic fields on short time scales during galaxy formation. Ideal AMR MHD models with Flash (Fryxell et al. 2000) by Sur et al. (2010) and Federrath et al. (2011b) demonstrated that an SSD can only be resolved in an accretion disc if the Truelove et al. (1997) criterion for resolving self-gravitating discs is satisfied with at least 32 cells, as shown in Fig. 4.

In this case, exponential growth can occur with timescales shorter than the orbital time of the disc. McKee et al. (2020) and Stacy et al. (2022) show growth of fields in primordial discs to values characteristic of modern star forming regions in simulations starting with cosmological structure formation and zooming in to disc scale. By characterising the numerical diffusivity, they found that the growth rates were consistent with the non-ideal MHD SSD theory of Xu and Lazarian (2016).

Whether modern protostellar accretion discs maintain their magnetic fields through the dynamo action of the MRI depends on how ionised they are. Dead zones where ambipolar or Ohmic diffusion dominate have fields in balance with magnetocentrifugally-driven winds, as suggested by local shearing-box models with Athena (Bai and Stone 2013), and then confirmed in a global disc model on a spherical-polar mesh using NIRVANA-III (Gressel et al. 2015). Active zones sufficiently ionised for the gas to fully couple to the magnetic field indeed have MRI-driven SSD action, as shown (Brandenburg et al. 1995) using uniform-grid, shearing-box, open-boundary simulations with the Stagger code (Nordlund and Stein 1990; Brandenburg et al. 1996), and in stratified, shearing-box simulations (Stone et al. 1996) with the ZEUS code (Stone and Norman 1992a, b).

### Kiloparsec scale

The structure of the magnetic field within the ISM on the kiloparsec scale can be separated into turbulent scales and the large-scale or MF. The separation scale is given by the effective 75–200 pc forcing scale of the turbulence driven by SN blast waves (Joung and Mac Low 2006; de Avillez and Breitschwerdt 2007) and stellar winds from massive stars (van Buren 1985). While the blastwave of a single SN evolves over a range of scales from its stellar radius to tens of parsecs, the effective forcing scale for the ISM turbulence is controlled by the collective action of multiple SNe combining to blow superbubbles (Korpi et al. 1999a; Joung and Mac Low 2006; de Avillez and Breitschwerdt 2007; Gent et al. 2013a; Hollins et al. 2017). Small-scale drivers from low-mass stellar winds, protostellar jets, planetary nebulae, and cloud dynamics also exist, but these are negligible compared to SNe and, to a lesser extent, the highest mass stars (Mac Low and Klessen 2004). The resulting turbulence can support a turbulent dynamo (or SSD), which amplifies the magnetic field exponentially.

The MF is organised by processes such as the differential rotation of galactic discs, spiral arms, and other metagalactic scale processes (Brandenburg and Subramanian 2005; Shukurov and Subramanian 2021). ISM turbulence, can, however, also be driven by large scale processes such as gravitational instability (Rafikov 2001), Kelvin–Helmholtz and other gas-dynamical instabilities, disc-halo circulation, and Parker instability.

#### SSD under ISM conditions

Numerical studies of SSD action in incompressible (Schekochihin et al. 2004) or weakly compressible (Haugen et al. 2004a) isothermal high-$\rm{Pr}_{\rm{M}}$ plasmas have been shown to lead to magnetic energies close to or even, at smallest scales, above equipartition. The ISM is however highly compressible and far from isothermal, significantly altering the response of the dynamo.

Numerical models of SSD action in the isothermal ISM, but considering compressiblity extending significantly beyond Mach 1, suggest that it produces and maintains magnetic fields of only a few percent of equipartition with the kinetic energy. This low saturation of the kinematic dynamo is evident for simulations of $\rm{Pr}_{\rm{M}}=1$ at high Mach number (e.g., Haugen et al. 2004b; Federrath et al. 2011a, 2014; Seta and Federrath 2021). Models of turbulence simulated at the value of $\rm{Pr}_{\rm{M}}>1$ relevant in the ISM show that the level of saturation increases with Pm, but still appears to reach an asymptotic limit well below equipartition (e.g., Schekochihin et al. 2002; Schober et al. 2012; Seta et al. 2020).

With isothermal turbulence driven by pure solenoidal forcing (Beattie et al. 2023) it is possible to reach around one third equipartition for $\rm{Pr}_{\rm{M}}=4$ at $\rm{Re}=500$. Seta and Federrath (2020) and Beattie et al. (2023) show that the SSD grows independently of the properties of the seed field in individual galaxies, while Garaldi et al. (2021) comes to the same conclusion using self-consistent cosmological simulations. SSD in a two-phase medium grows more slowly than in isothermal turbulence of comparable Mach number and Rm (Seta and Federrath 2022). It does appear that the SSD alone is insufficient to account for the observed strength of the turbulent magnetic field in galaxies.

**Fig. 5:**
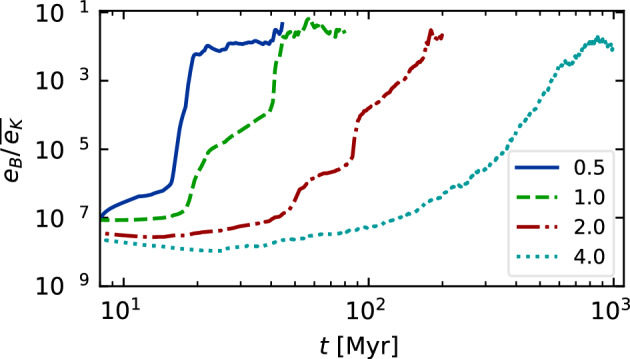
Magnetic energy scaled by time-averaged kinetic energy in a periodic box driven by SN explosions and radiative heating and cooling to produce a turbulent multiphase medium including explicit Laplacian resistivity and viscosity. Numerical resolution in parsecs is given in the legend. The timescale of exponential growth drops dramatically as resolution improves and Re$_{\rm{M}}$ increases. Image reproduced with permission from Gent et al. (2021), copyright AAS

With more realistic forcing in a multiphase ISM by SNe, at rates more relevant to spiral galaxies, but without any drivers for LSD, Balsara et al. (2004), Balsara and Kim (2005) and Gent et al. (2021) also find that the SSD saturates at a few percent of equipartition strength, but much faster than in isothermal models or the cooler two phase medium, as shown in Fig. 5. Because of the stochastic nature of the driving, this dynamo acts intermittently (Gent et al. 2021). Field growth is fastest in the hot phase where high sound speed facilitates high vorticity (Gent et al. 2023), but the strength at which the SSD saturates is lowest in the hot gas and increases in cooler phases. In particular, the turbulent flows of diffuse, inhomogeneous, hot gas in SN remnants and suberbubbles host the most rapid SSD (Gent et al. 2023) and (Kirchschlager et al. 2024, Fig. 1, third row).

In elliptical galaxies lacking differential rotation there is no driver for LSD, but SSD remains active, even though SN rates are much lower than in disc galaxies. The theoretical model of Moss and Shukurov (1996) anticipates a turbulent magnetic field similar in magnitude to those of spiral galaxies, while Seta et al. (2021) conclude from simulated observations of numerical turbulence that turbulent magnetic field must be an order of magnitude weaker.

Due to the random distribution of SNe and the multiphase structure of the ISM, the turbulent properties are highly anisotropic and intermittent. A thorough investigation of dependence of the turbulent magnetic properties on temperature phase and SN rate, and on galactic radial and vertical anisotropy remains outstanding. This would form the basis for modelling simulated observations of the magnetic structure of the ISM as well as constructing effective subgrid-scale models of MHD turbulence with which to explore large-scale magnetic effects.

#### Large-scale fields without LSD

To achieve the field strength observed in spiral galaxies, and also to comprehensively understand the anisotopy, structure and strength of the turbulent magnetic field, requires the presence of an LSD.

Some investigations have explored the evolution of a large scale field in a stratified ISM (e.g., de Avillez and Breitschwerdt 2005; Hill et al. 2012; Walch et al. 2015; Girichidis et al. 2016), but with an imposed initial uniform MF, which cannot be sustained in the absence of an MF dynamo mechanism. The turbulent field in these models arises primarily through tangling of the initial field. Comparisons between Balsara et al. (2004) and Gent et al. (2021) indicate that at the resolution applied in such models and using these codes, the SSD would take some 100 Myr to initiate and up to a gigayear to saturate, so most of these models are likely still in the unsaturated growth stage of the SSD.

#### SN driven LSD

To solve directly the evolution of the MF dynamo with turbulence driven by SN explosions Korpi et al. (1999) and Korpi (1999) model a stratified disc subject to rotation using a shearing box. Neither the SSD nor the LSD could be sustained because the model did not have the required resolution to reach magnetic Reynolds numbers sufficient to excite the SSD. With open vertical boundaries and a relatively low vertical extent the gas in these models is rapidly exhausted and overheated within a few hundred megayears. This is too soon to amplify the MF dynamo, which it is reasonable to expect be primarily hosted in the continuous warm phase of the ISM, rather than the isolated cold filaments or hot bubbles.

**Fig. 6:**
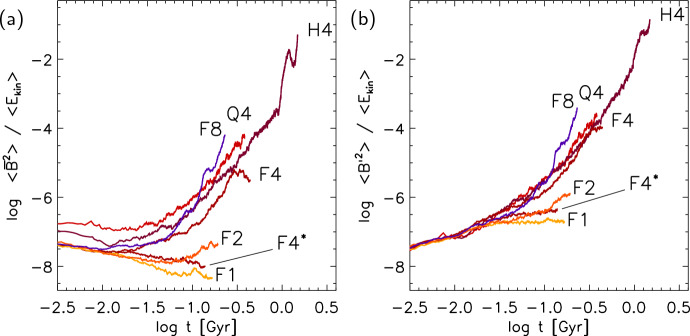
For all models of Gressel et al. (2008) **a** mean and **b** turbulent magnetic energy evolution. Labels Q, H and F denote SN rates of 0.25, 0.5 and $1 ~ \sigma _{\rm{Sn}}$, respectively, while the numbers give the angular velocity scaled by $~ \Omega _{\rm{Sn}}$. Image reproduced with permission from Gressel et al. (2008), copyright ESO

With the Nirvana code and double the grid resolution at (7.5 pc)$^3$, Gressel (2008) and Gressel et al. (2008) model an area 800 pc on a side extending ±2.1 kpc either side of the galactic disc. Exploring angular velocities ranging from $1~ \Omega _{\rm{Sn}}$ to $8~ \Omega _{\rm{Sn}}$, where the Sn subscript denotes the Solar neighborhood value, with and without shear and with SN explosion rates between $0.25~ \sigma _{\rm{Sn}}$ and $1~ \sigma _{\rm{Sn}}$, they find the LSD to be effective for $\Omega \gtrsim 2~ \Omega _{\rm{Sn}}$, see Fig. 6. In the absence of Coriolis forces, F4$^*$ in Fig. 6a, the shear produces no LSD. The efficiency of the LSD increases slightly as SN rate reduces (F4–H4 then Q4). The LSD does not saturate in these results, but it is clear that the timescale would exceed 1.5 Gyr for $\Omega \le 4~ \Omega _{\rm{Sn}}$.

Kim and Ostriker (2015) using a similar setup, but with the inclusion of a shearing radial boundary at $1~ \Omega _{\rm{Sn}}$, evolve their models beyond 850 Myr. Where the initial field is strong enough, the turbulent tangling and any residual SSD are sufficient to sustain an equilibrium total magnetic energy throughout, but for the model with a weak initial field the final state remains far below equipartition. This setup has the essential ingredients to model the LSD, but given the absence of any clear exponential growth it is very difficult to differentiate SSD from tangling and to exclude the effects of the imposed field on the results.

**Fig. 7:**
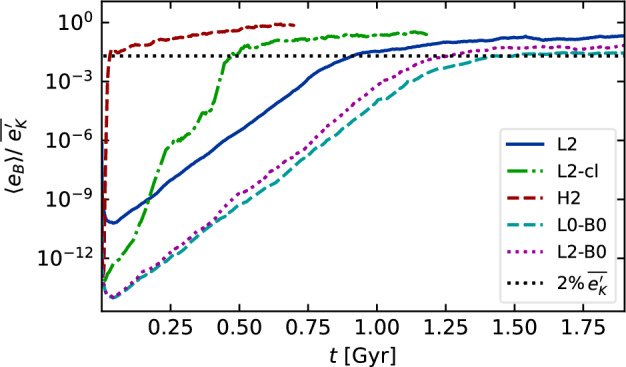
Evolution of total magnetic energy for all models from Gent et al. (2024), where L denotes resolution of $\delta x=4$ pc and H $\delta x=1$ pc. The suffix ‘cl’ denotes use of SN clustering and ‘B0’ the ongoing exclusion of an MF to isolate SSD activity. The numbers in the model labels denote $\Omega$ scaled by $~ \Omega _{\rm{Sn}}$. Image reproduced with permission from Gent et al. (2024), copyright the author(s)

With the Pencil Code, Gent et al. (2013a) almost double the resolution again at (4 pc)$^3$ to model a domain of horizontal area $(1.024 \text{ kpc})^2$ and extending 1.088 kpc either side of the disc. Applying differential rotation via the shear-periodic boundary, they confirm an LSD for $\Omega \ge 1~ \Omega _{\rm{Sn}}$. For a model with $\Omega =2~ \Omega _{\rm{Sn}}$, they obtain an MF dynamo with a growth rate of approximately 5 Gyr$^{-1}$, which is about twice that for the growth rate of the turbulent field. This would be incompatible with the results on the growth rates of the SSD in Sect. 3.3.1. Gent et al. (2013a), however, measure only the period 800 Myr to 1.05 Gyr, well after the SSD has saturated, but while the LSD remains an order of magnitude less than equipartition. To better understand the action of the SSD and LSD, Gent et al. (2024) study at this resolution, and also include resolution at (1 pc)$^3$. The volume averages of magnetic energy in each model are shown in Fig. 7.

With the growth and saturation properties of the SSD better defined by Gent et al. (2021, 2023), the effect of an active LSD on the SSD is isolated by systematically removing any horizontal MF from the components of the magnetic field (L2-B0 and L0-B0 of Fig. 7) in a stratified ISM with and without differential rotation. In the case with additional energy injected by the large scale shear from the differential rotation, the SSD grows slightly faster and saturates proportionately higher than in the non-rotating system. When the LSD is not excluded in the differentially rotating model (L2^2^), the SSD has a slightly lower growth rate during the kinematic phase. The LSD extracts energy from the SSD equivalent to the additional energy injected by the large scale shear, such that L2 and L0-B0 have the same kinematic growth rate. The kinematic SSD properties are therefore quite insensitive to the presence of the LSD.

The turbulent field for L2, however, does not stop growing after the SSD would saturate in the absence of the LSD, though growth slows as the LSD transitions into becoming the dominant mode of the dynamo. Surprisingly, perhaps, the magnitude of the growth of the turbulent field during this transition is orders of magnitude greater than that of the MF. Since the SSD appears to be saturated it seems tangling of the MF appears to be capable of amplification much greater than would typically be explained by mean-field theory, but could account for the observed high relative strength of the turbulent field.

While the SSD solutions are not converged for resolution as coarse as (2 pc)$^3$, as evident in the kinematic SSD stage for H2 and L2 in Fig. 7, it appears the LSD solutions may be sufficiently converged even for (4 pc)$^3$ resolution, which would be good news for future numerical study if confirmed. The effect of the SSD on the LSD requires further study.

From comparison of the evolving mean (Fig. 6a) and fluctuation (Fig. 6b) magnetic energy, it is evident that the SSD is absent for Gressel et al. (2008) while it is very active for Gent et al. (2013a) (Fig. 7). It is not clear why this would impede the progress of the LSD at low $\Omega$ for the former, while not in the latter. The LSD should be quite insensitive to the Rm in these ranges. It poses the question as to the extent the SSD may hinder or assist the action of the LSD. Gent et al. (2013a) do not obtain magnetic energy spectra with an obvious separation of scales between the mean and fluctuating magnetic fields, which instead show a single parabola. They do, however, show that a meaningful separation of scales can be defined with the fluctuating field occupying scales typically smaller than 250 pc and the large scale field having scales in excess of 700 pc.

**Fig. 8:**
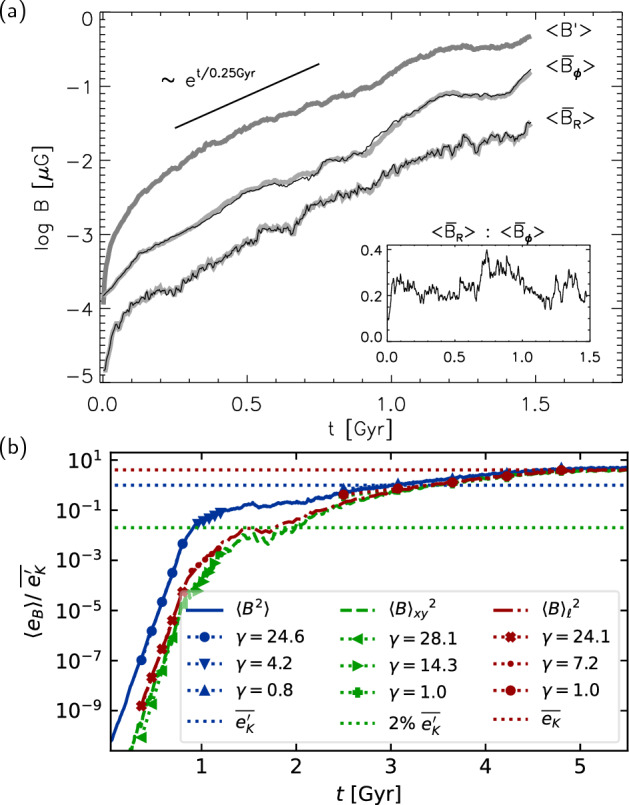
**a** Evolution of the horizontally averaged and the turbulent magnetic field strength for model H4 of Gressel et al. (2008) with SN rate $0.5 ~ \sigma _{\rm{Sn}}$ and angular velocity $4~ \Omega _{\rm{Sn}}$. **b** Evolution of the total magnetic energy and the mean field energy calculated from horizontal averages and convolution with a Gaussian kernel in model L2 of Gent et al. (2024). Images reproduced with permission, copyright [top] ESO and [bottom] the author(s)

Examples of decomposition of the mean and flucuation magnetic fields are displayed in Fig. 8 for the models of the galactic LSD by Gressel et al. (2008) and Gent et al. (2024). Figure 8a shows that for SN rate $\sigma =~ \sigma _{\rm{Sn}}$ the growth rate of the MF is proportional to $\Omega$ for $\Omega \ge 2~ \Omega _{\rm{Sn}}$. Gressel et al. (2008) do not find an MF dynamo for $\Omega =~ \Omega _{\rm{Sn}}$. However, Gent (2012) finds an MF dynamo is present at $\Omega =~ \Omega _{\rm{Sn}}$. Model F1 from Gressel et al. (2008) extends only to about 150 Myr, which may have been too short to dissipate transients from the initial azimuthal seed field. The linear dependence of the growth rate on $\Omega$ is consistent with the theoretical expectation of Brandenburg and Subramanian (2005) for a supercritical MF dynamo, which suggests the MF dynamo will be well supported in fast and even moderately rotating galaxies where the rotation curve is flat. In the range $0.25~ \sigma _{\rm{Sn}} \le \sigma \le ~ \sigma _{\rm{Sn}}$ the LSD is anticorrelated with $\sigma$.

**Fig. 9:**
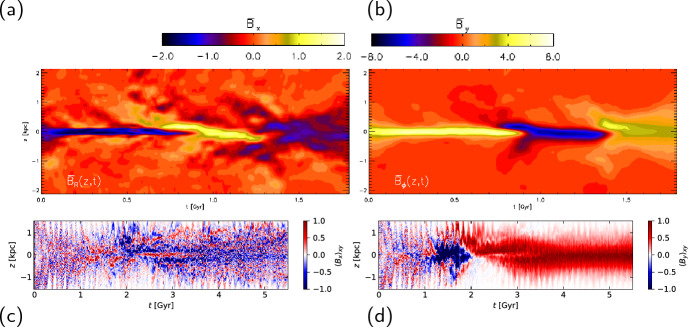
Evolution of the horizontally averaged mean magnetic field components, normalised by the maximum field strength at each time. From model H4 of Gressel et al. (2008) the field components **a** $B_r$ and **b** $B_\phi$ are shown. From model L2 of Gent et al. (2024) the field components **c** $B_x$ and **d** $B_y$ are shown. Both models are seeded by fields with maximum rms strength at the midplane $< 1$ nG, and grow to strengths of several microGauss

To understand how we might expect the dynamo generated mean field to appear near the galactic plane, we compare in Fig. 9 time-latitude diagrams of horizontal averages for model H4 from Gressel et al. (2008) and L2 from Gent et al. (2024). Model H4, in panels *(a)* and *(b)*, exhibits a quite regular field, subject to reversals with a period below 1 Gyr, and has quadrupolar structure. By contrast, model L2, in panels (c) and (d), initially has a rapidly fluctuating structure both in time and latitude due to the presence of a vigorous SSD. Well after the saturation of the LSD, the field becomes more regular. Early on there is more asymmetry with numerous reversals between different latitudes. As the field becomes more quadrupolar there is then a reversal around 2 Gyr, after which the field saturates to a steady azimuthally dominant field. The comparison between the models is most relevant up to about 2 Gyr, when both models are still subequipartition. It is noteworthy that the scale height of the magnetic field expands in the late stages when the strength of the midplane magnetic field becomes dynamically significant with respect to the kinetic energy. Whether the presence of reversals in the field is impacted by the rate of rotation, which differs by a factor of two here, the scale height of the disc, the SN rate, or the horizontal size of the domain requires further investigation.

Major open questions are prompted by these results. Within the first two Gyr the ratio of turbulent field to mean field remains substantially above 2 (Fig. 8), not inconsistent with observations. However by the time the LSD saturates, in conflict with observations, the mean field exceeds the strength of the turbulent field (see also Gressel et al. 2013a, their Fig. 2 and Gent et al. 2013b, their Fig. 6). For all simulations Rm and Re are many orders of magnitude lower than in the ISM, in which it is therefore easier to excite SSD and tangling, respectively. Is this sufficient to retard mean-field growth in the nonlinear LSD?

These numerical models tend towards solutions with pitch angles much closer to the azimuth than that observed. Is this due to weaker turbulence enabling the regular field to more easily align; is it a limitation of the shearing box approximation; or the limited horizontal aspect of the models reducing the degrees of freedom? In galaxy formation models the opposite seems to occur of failure to form a mean field.

#### Magnetic buoyancy instability

Another source of dynamo instabilities explored at these scales is the magnetic buoyancy instability (MBI) or when attenuated by cosmic rays, the Parker instability. A suitably stratified disc, with sufficiently strong parallel magnetic fields, has been initialized to examine the linear stage of the instability in an isothermal galaxy by Rodrigues et al. (2016). In such cases the mean magnetic field is imposed, presumably acquired by prior galactic dynamo, and the exponential growth of the velocity purturbations driven by the MBI drives a secondary dynamo. The resulting magnetic field has an expanded scale height and can attain superequipartition strength (Rodrigues et al. 2016). The growth rates during the linear stage of the instability obtained from the analytic linearised solutions of Giz and Shu (1993) are of order 20–30 Gyr$^{-1}$. Numerical solutions yield somewhat lower growth rates. When the numerical ratio of magnetic to gas pressure is high the growth rate of the linear instability is enhanced.

Including solid body rotation is anticipated analytically to dampen MBI growth (Ryu et al. 2003). However, Kim et al. (2001) find it makes little difference to the growth of the linear instability, though it can enhance the formation of turbulent structure in the magnetic field. The nonlinear state of MBI has been examined in the context of disc galaxies without (Tharakkal et al. 2023b) and with (Tharakkal et al. 2023a) rotation. MBI is characterised by an $\alpha$-effect with opposite sign of helicity to that typically driving the $\alpha \Omega$-dynamo (Tharakkal et al. 2023a; Qazi et al. 2024). When rotation is included MBI induces periodic reversals in the large-scale field (Tharakkal et al. 2023a) and the cosmic ray energy density is higher. Where the large-scale magnetic field is generated via a dynamo, the MBI has little effect on the stratified structure of the gas and cosmic rays, while still expanding the vertical scale height of the magnetic field, whereas when the field is imposed, it expands the scale height of the field and cosmic rays so far as to diminish their pressure support, resulting in a thinner gas disc during the nonlinear stage (Qazi et al. 2024). In Qazi et al. (2024) MBI and MF dynamos are investigated together by imposing an $\alpha$ effect into the equations, whereas MBI develops self-consistently. No rotation was included, nor its nonuniformities (differential rotation) were included, hence the dynamo was operating based on the imposed $\alpha$-effect alone. Such a dynamo is called $\alpha ^2$ as opposed to $\alpha \Omega$ which includes also differential rotation to provide a stronger amplification of the azimuthal component of the magnetic field. Therefore, the effect of differential rotation and the resulting $\alpha \Omega$-dynamo remains to be explored, as does the interaction between SN-driven turbulence and the MBI.

#### Magnetorotational instability

Investigating MRI-driven turbulence in a two-phase stratified ISM, Piontek and Ostriker (2005) find that, even without SNe, MRI is sufficient to excite strong turbulence in the diffuse ISM away from the midplane or in the outer galaxy where the ISM in the disc becomes more diffuse. MRI inhibits self-gravitating and thermal instabilites, likely retarding star formation in the outer galaxy (Piontek and Ostriker 2007). While turbulence can increase the proportion of the ISM in thermally unstable temperatures, the bimodal character of the ISM persists, consistent with the findings of Gent et al. (2013b) for the trimodal character in the presence of SN-driven turbulence. The strength of the magnetic field becomes somewhat independent of gas number density (Piontek and Ostriker 2005, 2007). However, decomposing the magnetic field between mean and turbulent contribution Evirgen et al. (2017) shows the turbulent magnetic field to correlate more with the hot phase and the MF with the warm and cold phases.

### Galactic scale

Schober et al. (2013) estimate the growth rate for an accretion-driven dynamo in a spherical protogalaxy to be

24 $$\begin{aligned} \Gamma \simeq k v_k \rm{ Re}^{1/2} \end{aligned}$$

for wavenumber *k*, velocity at that scale $v_k$, and Reynolds number Re, where the exponent is appropriate for Kolmogorov turbulence. They use this to analytically estimate that an accretion-driven small-scale dynamo can amplify a weak seed field to near-equipartition with the turbulent kinetic energy in tens to a few hundred megayears. These are almost certainly overestimates of the time required for these fields to grow, since equipartition level small scale fields already grow in the first protostellar accretion discs, as described in Sect. 3.2.

#### Mean-field (MF) models

There exist proof-of-concept nonlinear MF models employing the full dynamic quenching formula with various types of helicity fluxes. For example, the studies of Shukurov et al. (2006) and Sur et al. (2007) investigate galactic fountain-driven fluxes and the latter study also investigates helicity fluxes resulting from anisotropic turbulence due to the presence of shear, a scenario proposed by Vishniac and Cho (2001). Also diffusive helicity fluxes have been included by Chamandy et al. (2014). All these studies were able to demonstrate that including helicity fluxes results in LSD saturating at the equipartition value with turbulence being excited and maintained. Also, evidence for the heuristic nonlinearity (Eq. (16)) producing results consistent with the more complete quenching models was found (e.g., Chamandy et al. 2014). This is reassuring, as both old and new MF models still often use the heuristic nonlinearity (e.g., Liu et al. 2022). Chamandy et al. (2013a) apply a mean-field model (Chamandy et al. 2014) additionally including non-locality in time and forcing by spiral arms into the $\alpha$-effect. As a result, they see the emergence of prominent magnetic spiral arms, which has potential to resolve the difficulty in exciting and maintaining non-axisymmetric modes.

#### MHD models

**Fig. 10:**
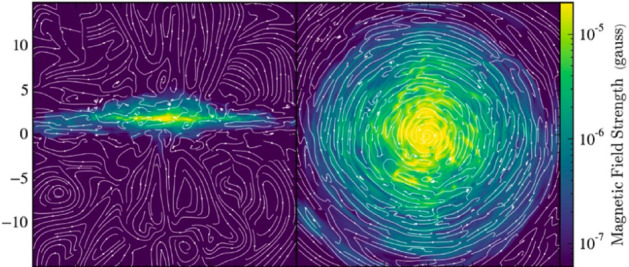
Magnetic field strength projected edge-on and face-on in an isolated disc galaxy. Directed streamlines of magnetic field are shown in white. The length scale is given in kiloparsecs on the left axis. Image reproduced with permission from Butsky et al. (2017), copyright AAS

The interrelation between the SSD and the LSD is a key question at the galactic scale. The physical timescale for the SSD is far shorter than for the LSD. However, insufficient numerical resolution can dramatically slow the action of the SSD (Gent et al. 2023), causing the two timescales to apparently overlap in numerical simulations. Nevertheless, numerical simulations do produce toroidal fields that generally reproduce the observed topology, as shown, for example, in Fig. 10.

***Small-scale dynamo***

**Fig. 11:**
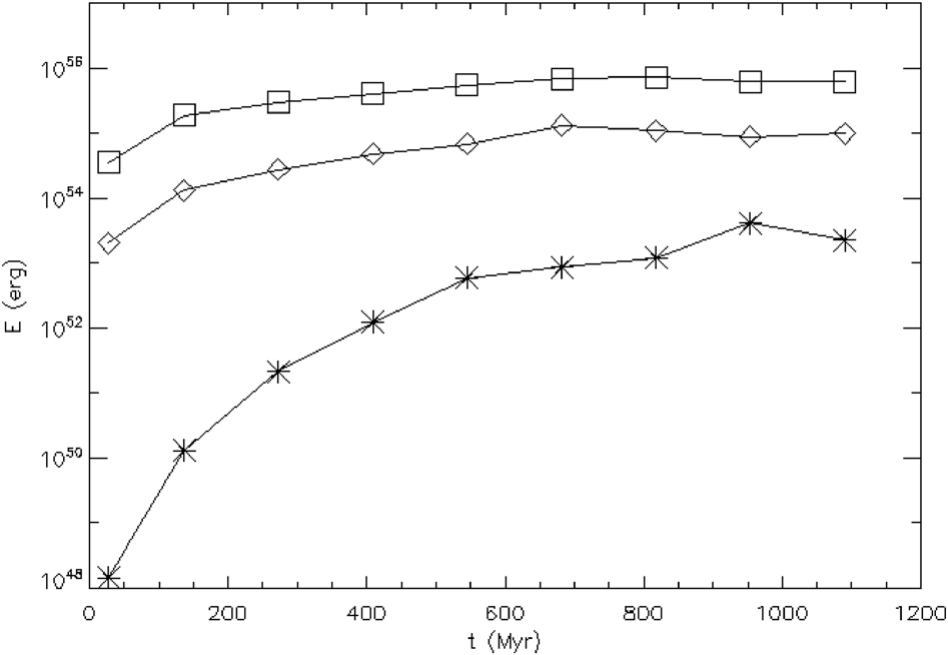
Magnetic energy (asterisks) in disc for a dwarf galaxy simulation at redshift two without stellar feedback at 26 pc resolution compared to kinetic energy (squares) and thermal energy (diamonds). including SN feedback at the finest resolutions given in the legend. Exponential growth with the given time scales is shown by the black lines. Image reproduced with permission from Wang and Abel (2009), copyright AAS

The presence of SSD in global galaxy models has been demonstrated using two primary diagnostics: the exponential growth of magnetic energy and the agreement of the power spectrum of the field with the $k^{3/2}$ prediction of Kazantsev (1968). The first global MHD models of field development in a disc galaxy were performed by Wang and Abel (2009) and Kotarba et al. (2009), neglecting turbulence driven by stellar feedback. These works already demonstrated that the turbulence generated by gravity and differential rotation in galaxies is sufficient to produce exponential field growth to a few percent of equipartition, as shown in Fig. 11, although at a rate limited by the low numerical resolution and turbulence strength. Kotarba et al. (2009) further demonstrated that constraint of magnetic helicity by evolution of the field using Euler potentials (Price and Bate 2007) suppresses dynamo activity.

The first diagnostic, exponential growth of magnetic energy to a few percent of kinetic energy in global galaxy simulations has been confirmed by multiple groups in the last decade. Models without explicitly modeled feedback-driven turbulence include Pakmor and Springel (2013), Rieder and Teyssier (2016), Pakmor et al. (2017), Rieder and Teyssier (2017), Steinwandel et al. (2020) and Pfrommer et al. (2022). In most of these models, feedback is assumed to contribute to a turbulent pressure variable but the only flows contributing to an SSD are produced by gravitational dynamics of the galactic gas or large-scale galactic winds. A number of studies without explicit feedback did not explicitly report the exponential growth of magnetic energy, but rather that of related but not identical quantities such as the average magnetic field strength, including Pakmor et al. (2014), Steinwandel et al. (2019) or the median magnetic energy density Pakmor et al. (2017). Models that do include stellar feedback, driving turbulence at smaller scales and resulting in faster magnetic energy growth, include Su et al. (2017), Butsky et al. (2017), Ntormousi et al. (2020) and Martin-Alvarez et al. (2022).

**Fig. 12:**
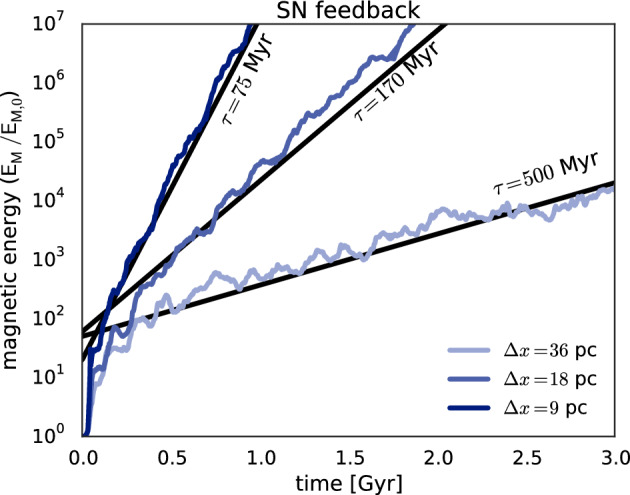
Magnetic energy in domain for a dwarf galaxy simulation including SN feedback at the finest resolutions given in the legend. Exponential growth with the given time scales is shown by the black lines. Image reproduced with permission from Rieder and Teyssier (2016), copyright the author(s)

Rieder and Teyssier (2016) clearly demonstrated the importance of numerical resolution in full galaxy models, as shown in Fig. 12. Note that these are still low resolutions and thus slow growth rates compared to those reached in kiloparsec-scale models (see Sect. 3.3.1), where growth time scales drop to under a megayear (Fig. 5 at 0.5 pc resolution. Martin-Alvarez et al. (2022) emphasised the importance of uniformly resolving galactic discs rather than allowing resolution to follow density to correctly capture the growth of magnetic fields in lower-density regions where they grow faster.

**Fig. 13:**
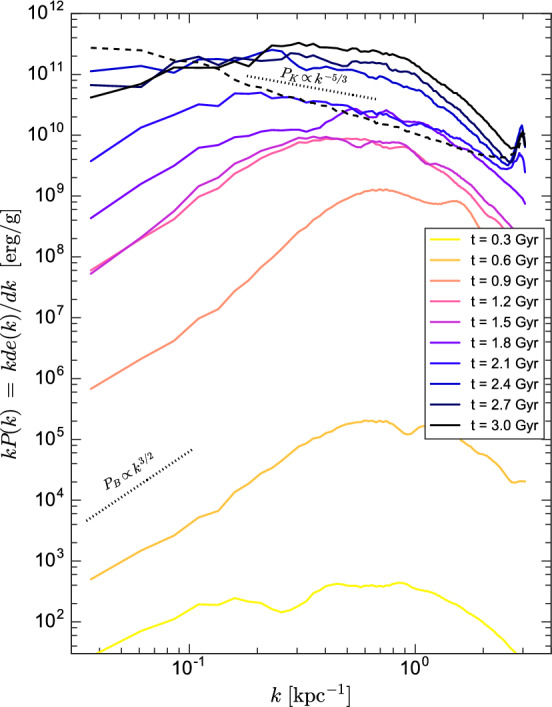
Power spectrum of magnetic energy at listed times in a model of an isolated galactic disc. These spectra show the Kazantsev slope at large scales, and a peak that occurs at increasing scale over time, as expected from a saturating SSD. The data and the guiding lines (*dotted lines*) have been regularised by *k*. The kinetic power spectrum at the final time is also shown (*dashed line*), with a slope steeper than expected for incompressible Kolmogorov turbulence, consistent with the compressible turbulence in this galaxy. Image reproduced with permission from Butsky et al. (2017), copyright AAS

The second diagnostic of SSD action is the development of Kazentsev spectra in models followed by the shift of the peak in magnetic energy from the dissipation scale to longer wavelengths as saturation sets in at the dissipation scale, as shown, for example, in Fig. 13. This result has been found in full-galaxy models by Rieder and Teyssier (2016, 2017), Rieder and Teyssier (2017), Butsky et al. (2017), Pakmor et al. (2017), Martin-Alvarez et al. (2018), Steinwandel et al. (2019), Martin-Alvarez et al. (2022) and Pfrommer et al. (2022). Steinwandel et al. (2019) and Ntormousi et al. (2020) noted that after saturation, an Iroshnikov spectrum with magnetic energy proportional to $k^{-3/2}$ appears, while Pfrommer et al. (2022) argues that the saturated spectrum is closer to Kolmogorov with a $k^{-5/3}$ dependence, although in an accretion-dominated model of a primordial galaxy.

Martin-Alvarez et al. (2018, 2022) and Ntormousi et al. (2020) using AMR and Steinwandel et al. (2020) using SPH explicitly analysed SSD and LSD in global galaxy simulations including a subgrid model of SN feedback (Springel and Hernquist 2003; Dubois and Teyssier 2008). They all compared models with and without feedback and found that both showed the same exponential growth during the first 0.5 Gyr. At the resolution of these models, this is the expected timescale for the action of the SSD, suggesting that the primary driver of the turbulence required for SSD may be accretion (Klessen and Hennebelle 2010) rather than SNe in these models that include circumgalactic gas.

***Large-scale dynamo***

**Fig. 14:**
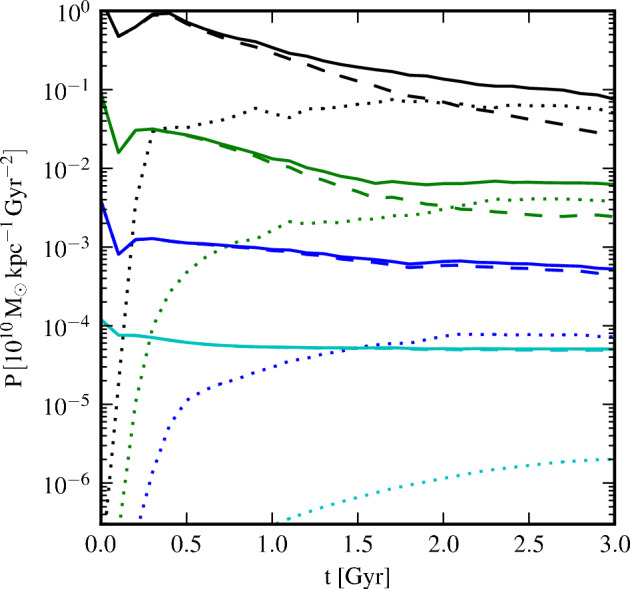
Magnetic (dotted lines), thermal (dashed lines), and total (solid lines) pressure for halos with masses of $10^9$ to $10^{12}\,M_{\odot }$. All pressures are volume-averaged over the galactic discs. These models show LSD behavior whose relative and absolute saturation strength depends strongly on potential well depth. Image reproduced with permission from Pakmor and Springel (2013), copyright the author(s)

Evidence for the presence of the LSD in global models including SSD has only started to be presented within the last several years. An early paper that likely showed LSD action, but did not discuss it explicitly, is Pakmor et al. (2016). They compared the growth of volume-averaged magnetic pressure to that of total and thermal pressure in galaxies of halo mass varying from $10^9$ to $10^{12}\,M_\odot$, so from dwarf to Milky Way sizes. As shown in Fig. 14, all of these galaxies showed at least modest further growth in magnetic pressure after saturation of the SSD. However, the saturation of this apparent LSD depended strongly on the halo mass, with the smallest, slowest rotating halos with the shallowest potentials having magnetic pressures more than an order of magnitude below the thermal pressure, while the Milky Way mass halo had magnetic pressure exceed thermal pressure.

**Fig. 15:**
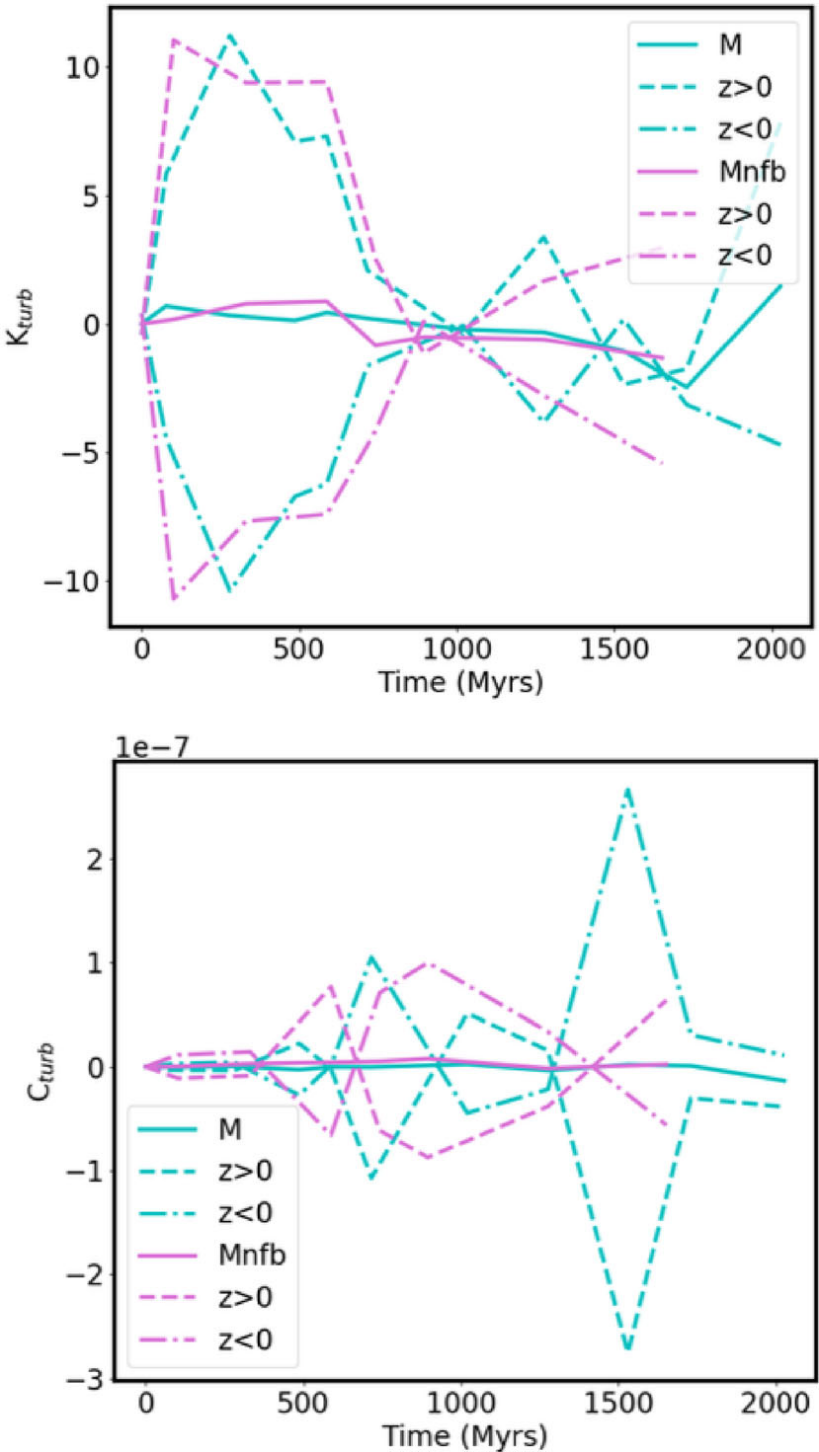
Integrated turbulent kinetic and current helicities over time above and below the galactic midplane in a global AMR model embedded in a halo. The symmetric sign reversals suggest an unsaturated dynamo. Image reproduced with permission from Ntormousi et al. (2020), copyright by ESO

As helical turbulence is believed to be one of the most important turbulent ingredients for an LSD, Ntormousi et al. (2020) directly measured the turbulent kinetic and current helicities (see Eqs. (18) and (19)). A median filter with 390 pc (8 cell) size was used to separate turbulent and mean components of the velocity and magnetic field. In these AMR models, the magnetic energy remained below 0.1% of the turbulent kinetic energy, so these are representative of an unsaturated dynamo. This is supported by the multiple sign reversals across the midplane seen in Fig. 15.

**Fig. 16:**
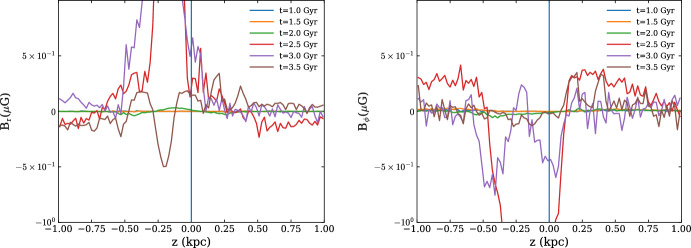
Vertical dependence of radial *(left)* and toroidal *(right)* magnetic fields at times given in the legend for an SPH global model. Antisymmetry around the midplane suggests dipole structure, while symmetry suggests quadrupolar structure. Some indications of both structures are seen at different times, but the full structure is more complex, and influenced by the buildup of a magnetic tower flow. Image reproduced with permission from Steinwandel et al. (2020), copyright the author(s)

Steinwandel et al. (2020) and Ntormousi et al. (2020) both found that the classical dipolar and quadrupolar field structures do not fully describe the large-scale field resulting from their models of LSD in global galaxies, with a rather more confused pattern probably consisting of multiple eigenfunctions growing simultaneously instead appearing, as shown for example in Fig. 16. Similar results were found by Pakmor et al. (2018) and Reissl et al. (2023) while computing Faraday rotation measures, as discussed in Sect. 3.6 below.

### Cosmological scale

The inclusion of the cosmological environment in models of dynamos in global galaxies allows a more self-consistent treatment of accretion on to galaxies and the influence of interactions with neighbors. The cost is a low numerical resolution compared to the models of single galaxies discussed in the previous section. However, even with SPH mass resolutions as high as $2\times 10^7\,M_\odot$, Beck et al. (2012, 2013) demonstrated that an SSD produces exponential field growth during cosmological halo formation, accretion, and merging even though disc formation was not resolved. Radiative cooling balanced by star formation driven feedback (implemented with a star formation threshold of only 0.13 cm$^{-3}$ in these low resolution models) produced the fastest growth rates, with e-folding times of order $10^7$ yr, and equipartition being achieved with the turbulence. Saturation occurred with field strengths of $10^{-5}$ G at the centers of halos.

More recent simulations have followed the formation and evolution of star-forming galaxies by zooming in to cosmological simulations, improving the numerical resolution within galaxies in order to resolve dynamo action there. Pakmor et al. (2014) used the Voronoi-mesh code AREPO to model a Milky-Way sized galaxy with mass resolution of $5\times 10^4\,M_\odot$ and a gravitational softening length of 340 pc, while Rieder and Teyssier (2017) used the AMR code RAMSES to model a dwarf galaxy down to redshift $z = 4$ with most refined resolution of 22.5 physical pc (requiring successive refinements during expansion of the universe). These works found that an SSD drives initial exponential field growth. The small-scale turbulence is initially produced by chaotic accretion, and then subsequently by star formation. In these works, increasing the resolution increases the growth rate of the SSD as expected. After saturation of the SSD, they find linear growth of the LSD in the disc, while fields in the galactic center remain at the value produced by saturation of the SSD absent significant rotation.

**Fig. 17:**
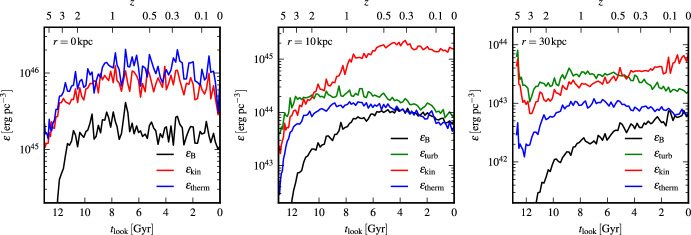
Median energy densities over time drawn from the 30 zoom-in models of the cosmological Auriga simulations. Measurements are performed in rings at the given radii. At 10 kpc galactocentric radius, the magnetic field only approaches equipartition with the turbulent kinetic energy after 10 Gyr of evolution. Image reproduced with permission from Pakmor et al. (2017), copyright the author(s)

Larger scale efforts include the Auriga simulations (Grand et al. 2017), a set of 30 zoom-in models using AREPO of Milky Way mass haloes drawn from the dark matter only version of the Eagle model (Schaye et al. 2015) at the same mass resolution of $5\times 10^4\,M_\odot$ used by Pakmor et al. (2014). Although these models still do not capture the physical growth rate of the interstellar SSD (Sect. 3.3), they do follow the galactic LSD acting on the fields generated by halo-scale SSD action during the galaxy formation process. As the galactic LSD appears insensitive to resolution in this regime (Sect. 3.3), the structures modeled in this way may reflect physical processes and not just numerical dissipation (Fig. 17).

Perhaps the highest resolution cosmological zoom-in model to date is described by Martin-Alvarez et al. (2023), who have simulated a dwarf galaxy in its cosmological context with 7 pc finest grid resolution using the RAMSES AMR code. The MHD runs were terminated at redshift $z = 3.5$ for reasons of computational cost. At that time, fields of 2–5 $\upmu$G had built up in their discs, consistent with observed values of $z = 0$ dwarf galaxies (Chyży et al. 2011). Unfortunately, these models have not yet been analysed in any detail for their dynamo properties.

None of these models has a spatial resolution much better than the highest resolution model shown in Fig. 12, with 9 pc resolution, which has an exponential growth time of 75 Myr, exceeding the Jeans time for gravitational collapse in these galaxies of a few tens of megayears, and a large fraction of their rotation time. However, actual growth times of only a few megayears are predicted by the models with 0.5 pc resolution shown in Figs. 5 and 7. Thus, fields with strengths of a few percent of equipartition should be present very shortly after gravitational collapse and star formation have set in. Global models of modern galaxies with typical resolutions that start with small seed fields and only grow strong fields over large fractions of a gigayear can thus only be interpreted as physics experiments exploring the behavior of dynamos. However, they can not be intepreted fruitfully to understand the astrophysical evolution of galaxies in the presence of magnetic fields, because of the extended period of weak field present in these models that should not occur in any physical scenario including cosmological growth of galaxies.

### Observational comparisons

The correspondence between spiral structure in mean magnetic fields and spiral structure in stars and gas is observed to vary (e.g., Beck 2015; Bittner et al. 2017), with the strongest mean fields often appearing in the interarm regions, anticorrelated with the stars and gas. This behavior is reproduced at some times by the global model of a Milky-Way mass galaxy including a circumgalactic medium by Steinwandel et al. (2019). They hypothesise that the stronger turbulence in the lower-density, hotter interarm regions leads to faster field growth there.

**Fig. 18:**
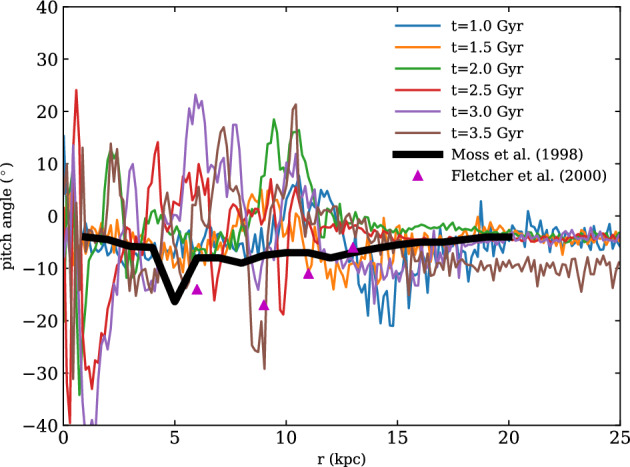
Radial profiles of average pitch angle of magnetic field over time for a Milky Way mass galaxy, at times given in the legend. Negative pitch angles consistent with observations predominate, although positive pitch angles occur in regions heavily perturbed by magnetic tower outflows. The *purple triangles* show observations of M31 by Fletcher et al. (2000); these are consistent with the Milky Way value of $-15^{\circ }$ at the Solar circle. The *black line* is from a dynamo model of M31 by Moss et al. (1998). Image reproduced with permission from Steinwandel et al. (2020), copyright the author(s)

The pitch angle of the spiral structure in magnetic fields is another point of comparison between global simulations and observations. Models show a great deal of scatter in the pitch angle, as shown in Fig. 18, with shallow negative pitch angles consistent with observations being frequent, but by no means universal. The situation is further confused by accretion, outflows, and interactions with other galaxies, all of which can distort the magnetic field sufficiently for the pitch angle to become positive or strongly negative.

Carteret et al. (2023) used a shearing box model of a section 0.8 kpc on a side of a galactic disk to measure the structure of Faraday rotation and polarisation due to dynamo-derived fields. They found that different assumptions about the thermal electron density distribution, not explicitly modeled in their simulation absent radiative transfer of ionising radiation, drive leading order variation in the magnitude and scale distribution of the rotation measure for the same magnetic field structures. Anisotropic structure in the rotation map occurred under both assumptions, though.

**Fig. 19:**
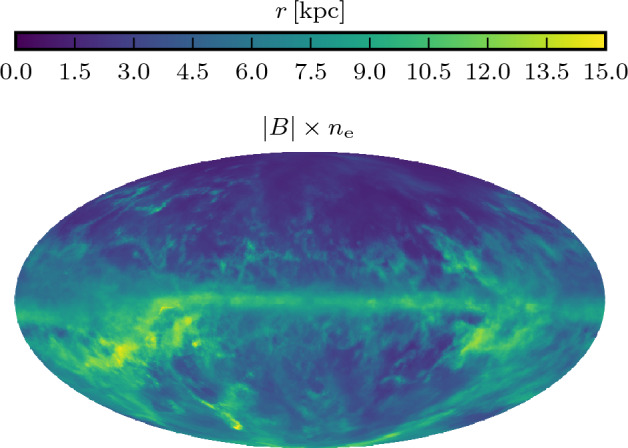
Average depth along the line of sight of contributions to the product of the magnitude of the magnetic field and the density of thermal electrons measured by rotation measure. The simulated observations are performed for one of the Auriga zoom-in models of galaxies in a cosmological simulation. Particularly at high latitudes, the dominant contribution comes from the local neighborhood rather than the disc as a whole. Image reproduced with permission from Pakmor et al. (2018), copyright the author(s)

**Fig. 20:**
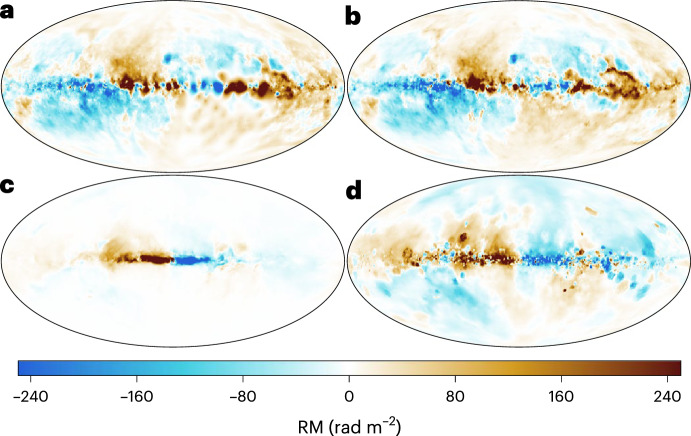
Comparison between the all-sky rotation measure maps derived by **a** Oppermann et al. (2012) and **b**Hutschenreuter et al. (2022) to the rotation measure map from **c** an unmodified Auriga zoom-in model of a galaxy, and **d** from the same model and viewpoint but with a subgrid model of ionising radiation from star clusters determining the thermal electron distribution. The additional small-scale structure reproduces the statistical small-scale structure distribution of the observations far better. Image reproduced with permission from Reissl et al. (2023), copyright the author(s)

Pakmor et al. (2018) and Reissl et al. (2023) modeled observed Faraday rotation on the sky as seen from within one of the galaxies from the Auriga cosmological zoom-in simulations (Grand et al. 2017) at roughly the Solar circle. These models were compared to the observed distributions derived by Oppermann et al. (2012) and Hutschenreuter et al. (2022) from measurements towards extragalactic sources. Pakmor et al. (2018) found agreement on the strength and qualitative distribution of intermediate to large scale structure in the distribution, but found much less small-scale structure than in the observations. They found that the structure within a few kiloparsecs dominates the Faraday rotation, particularly at high latitudes, rather than any global dynamo-formed dipole or quadrupole field, as shown in Fig. 19. Reissl et al. (2023) used cluster population synthesis and explicit transfer of ionising radiation (Pellegrini et al. 2020) to model the distribution of thermal electrons down to a much smaller scale, allowing polarised radiative transfer calculations from a vantage point within a superbubble at roughly the Solar radius. Consistent with the results of Carteret et al. (2023), they find that the addition of the subgrid model of cluster ionisation dramatically improves the agreement of the model with the observations at smaller scales as shown in Fig. 20 and quantified in the paper.

**Fig. 21:**
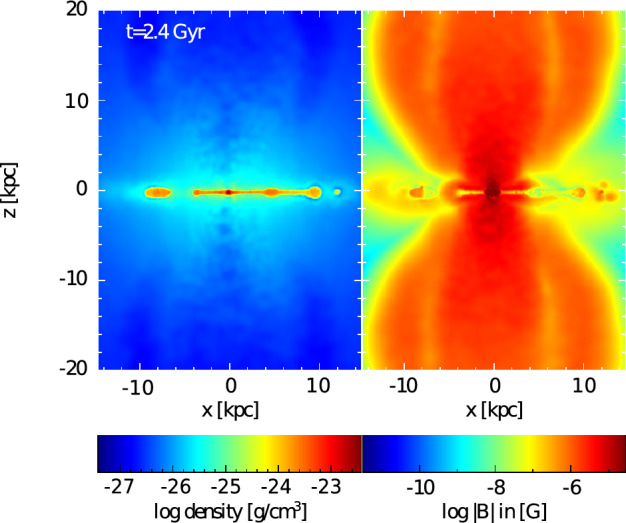
Slices of density and magnetic field magnitude for Milky-Way mass models including circumgalactic medium structure after 2.4 Gyr of evolution. The magnetic tower outflow driven by magnetic pressure produces X-shaped structures in the circumgalactic medium similar to those observed. Image reproduced with permission from Steinwandel et al. (2019), copyright the author(s)

A strong magnetic tower flow (Fig. 21) with a velocity of a 400–500 km s$^{-1}$ driven by the dynamo-driven increase of magnetic pressure in the center of the modeled galaxy occurs in the model of Steinwandel et al. (2019). This structure appears consistent with the X-shaped magnetic field structures seen emerging from disc galaxies in observations of radio polarisation (e.g., Veilleux et al. 2005). However, this is a transient effect that lasts only a few hundred megayears in the model, raising the question of how such a structure can be commonly observed in modern galaxies.

**Fig. 22:**
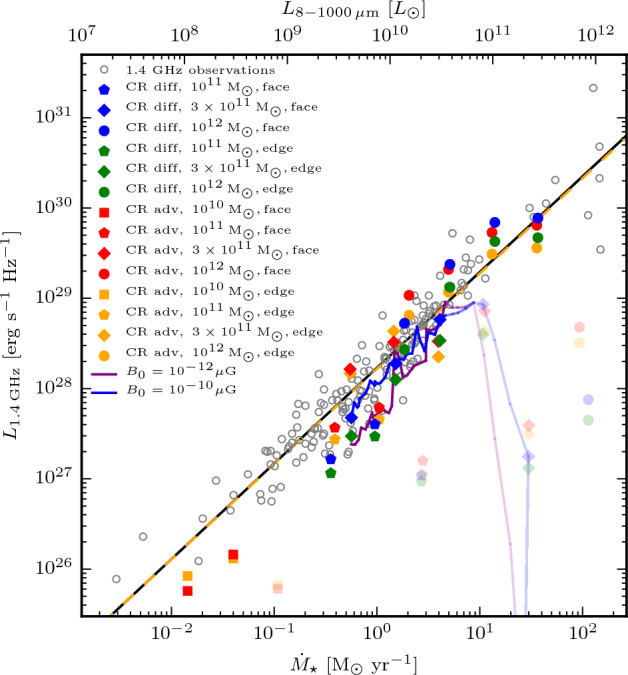
Comparison of observations of the correlation between radio synchrotron luminosity at 1.4 GHz and far infrared luminosity at 8–1000 $\upmu$m *(open circles)* to luminosities computed from simulations of accretion dominated galaxies with the Voronoi mesh code AREPO. The semi-transparent symbols and tracks show results from unsaturated dynamos and their evolution, while the solid colors represent fully saturated dynamos. Models with only cosmic ray advection are labeled “CR adv” in the legend, while models also modelling cosmic ray diffusion are labeled “CR diff”. The legend also gives halo masses and orientation for the different galaxies. Image reproduced with permission from Pfrommer et al. (2022), copyright the author(s)

A detailed comparison of the interaction between fields generated by the SSD and cosmic rays in an accretion dominated galaxy to the correlation between far-infrared and radio synchrotron luminosities was presented by Pfrommer et al. (2022). Figure 22 shows that the correlation can be reproduced by fully saturated SSDs in these galaxies, a result that is robust to the details of the modelling of cosmic ray travel and the orientation of the galaxies. Carteret et al. (2023) examined polarisation of synchrotron emission in their shearing box model with an assumed distribution of cosmic rays. They showed that the cosmic ray distribution and the field both matter for the synchrotron emission, and that the observed structure further depends on the wavelength of observation.

Another comparison between simulations including cosmic rays and observables was done by Ponnada et al. (2022). They compared synthetic rotation measures to observational constraints from the circumgalactic medium around galaxies. They found rotation measures consistent with observed upper limits, but low enough that they would not be detected with current instruments, suggesting that improved observations will be needed to fully constrain these models.

## Properties of turbulence

Numerical models have been used to extract dynamo-relevant properties of turbulence. Such studies have two purposes. Firstly, comparisons with analytical theories can be made. Secondly, some ill-known parameters and properties can be deduced, which can be vital to constructing simpler and less resource-demanding numerical approaches.

### Retrieval of turbulent transport coefficients

Semi-analytic approaches (see, e.g., Ferrière 1998, and references therein) showed early on that isolated SNe cause only weak turbulent effects relevant for the dynamo, while superbubbles could produce more significant effects in the required range for producing a dynamo number above the critical one needed for LSD action. These models, however, excluded SN interactions, such as colliding shock fronts, where nonlinear interactions, such as generation of vorticity through the baroclinic effect (arising when temperature and entropy gradients are misaligned) or vortex stretching, have been reported to generate significant amounts of vorticity (see, e.g., Korpi et al. 1999; Käpylä et al. 2018). To study such effects, full MHD models are needed.

**Fig. 23:**
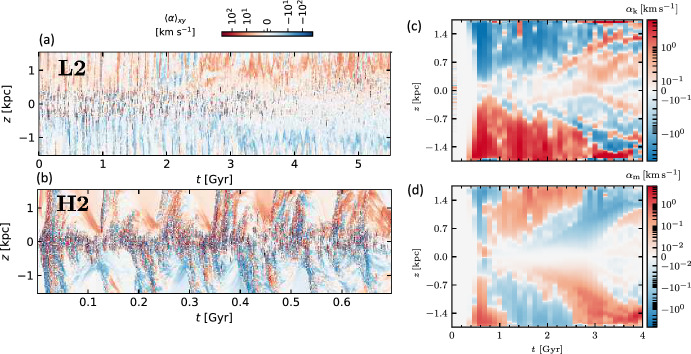
Time-latitude diagrams of horizontally averaged $\alpha$-effect for a SN-driven model with twice the galactic rotation and shear rate from **a** a lower-resolution model integrated up to the saturation of the dynamo and **b** a higher-resolution model with a far shorter time extent, but illustrating the detailed time evolution. Adapted from Gent et al. (2024). On the right we show **c** the kinetic $\alpha$ value and **d** the magnetic $\alpha$ value from a Parker-unstable system without SNe, but with galactic rotation rate. Adapted from Tharakkal et al. (2023a)

The most straightforward way of estimating the turbulent inductive and diffusive effects from numerical simulations is to use kinetic and magnetic helicity and turbulent intensity as a proxy for them (see Eqs. (18) and (19)). To date, this method has only rarely been implemented to diagnose the full MHD simulations. Recently, Gent et al. (2024) computed the proxy for the $\alpha$ effect this way in their SN-forced, stratified, rotating and shearing local model, exciting both SSD and LSD, and ran their lower-resolution models until the saturation of the LSD (see Fig. 23a and b). In the growth stage of the LSD the kinetic $\alpha$ dominates, is smooth and of the expected sign ($+$ in the upper and − in the lower part of the disc) in regions of more laminar inflows caused by cooling. On the other hand, it is strong and spatially incoherent with undetermined sign in the strong outflows caused by clustered SN activity. The unexpected sign of $\alpha$ in the latter case seems to originate from the opposite disc plane, which indicates that some non-trivial helicity fluxes might occur across the midplane of the disc. The magnetic $\alpha$ appears to have two clear roles: to reverse the sign of the $\alpha$ effect during a reversal of the azimuthal magnetic field at approximately 2 Gyr, and to saturate LSD growth in the warm gas concentrated around the midplane.

Tharakkal et al. (2023a) derived the $\alpha$ contributions in the same way from a Parker unstable stratified disc otherwise similar, but without SNe and Milky Way solar neighborhood rotation and shear rates (Fig. 23c and d), while Gent et al. (2024) had twice the Solar neighborhood values. A noteworthy feature, in comparison to the SN-driven system in panels Fig. 23a and b is that the kinetic $\alpha$ effect is systematically of the opposite sign (up to 2 Gyr). The smooth parts of the $\alpha$ effect in the different cases are roughly of the same magnitude, while significantly stronger only in the SN bursts and the resulting outflows. The higher rotation and shear rates in Gent et al. (2024) most likely also enhance the SN-driven $\alpha$ magnitudes, so they might, in fact, be rather equal in a consistently similar system. The early change in sign of the magnetic part of the $\alpha$ effect is similarly relatable to a reversal of the azimuthal magnetic field in the Parker-unstable case. Also, in this case the LSD is saturated by the growing magnetic contribution of the opposite sign. Comparing these two studies it is evident that the Parker-instability induced dynamo is of significance and operates in a markedly different way than an SN-driven dynamo, while both are expected to occur in the SN-active part of galactic discs. Hence, future numerical studies including both these mechanisms is of great importance. Such studies, however, require large enough domains also in the SN-forced models to capture the Parker unstable magnetic loops.

**Fig. 24:**
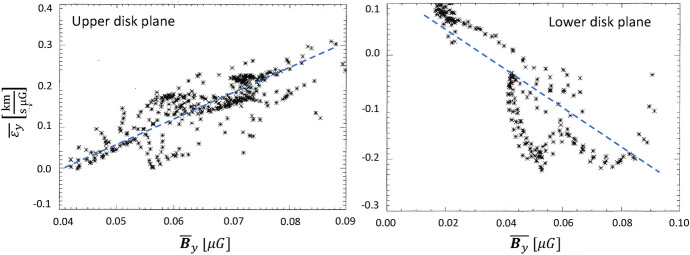
The mean azimuthal magnetic field versus the mean azimuthal electromotive force. Averages are taken over space and over a 10 Myr time interval, separately for the *(left)* upper and *(right)* lower disc plane of the Cartesian shearing box simulation. This time period roughly corresponds to the turnover time in the local simulation domain. The blue dashed lines show $\alpha _y = \pm 6$ km s$-1$ correlation coefficient from the relation ${\overline{{{{\mathscr {E}}}}}}_y=\alpha _y \overline{{{\varvec{B}}}}$. Image adapted from Korpi (1999)

Another numerical method to retrieve a limited set of the turbulent transport coefficients is the imposed field method. It relies on the Taylor expansion of the mean emf according to Eq. (11) under the assumption that all higher order terms than the linear term containing the $\alpha$ coefficients vanish; this requires the imposed magnetic field to be uniform. One imposes a field in some direction, and then measures the mean electromotive force components and the mean magnetic field, and finally solves for the relevant $\alpha$ tensor component(s). The problem is that non-uniform magnetic fields can be induced, leading to the formation of currents relatively quickly. These currents cause the breakdown of the method unless the magnetic field is reset at suitable time intervals (see, e.g., Ossendrijver et al. 2002; Hubbard et al. 2009). This method has not been applied very extensively in the context of galactic dynamos, although early attempts (Fig. 24) resulted in a successful measurement of the main $\alpha$ effect component generating the mean radial field from the azimuthal one (Korpi 1999). There was no explicit imposed field applied, as the system itself was dynamo active, and resulted in the amplification of mean fields. Due to poor resolution, the dynamo was near its critical Re$_{\rm{M}}$ for being active. Therefore, the field never reached high strength due to the limited duration of the simulation runs, and thus remained in the kinematic regime. Indications of an $\alpha \Omega$ dynamo with an $\alpha _y$ coefficient of roughly $\pm 6\,\mathrm {km\ s}^{-1}$ were found. This was a factor of a few in excess of the nearly contemporary semi-analytic findings that neglected SN interactions, thus indicating that the SNe are, indeed, important.

Brandenburg et al. (1995) studied accretion disc dynamos driven by MRI in a Keplerian shearing box model. They found a self-sustained dynamo, and applied a similar method to measure the $\alpha _y$ component as Korpi (1999). As a result, a clear correlation between the mean emf and the azimuthal magnetic field was again found, but the sign of $\alpha _y$ was the opposite: negative in the upper and positive in the lower hemisphere, similar to the Parker-unstable system of Tharakkal et al. (2023a). This suggests that dynamos driven by magnetic buoyancy might all have this feature in common. Also galactic discs are susceptible to MRI (Piontek and Ostriker 2005, 2007) although their rotation laws are not Keplerian, but typically flat. Therefore, although the Brandenburg et al. (1995) results are not directly applicable, they could indicate that multiple different dynamo mechanisms could be at play in galaxies, if SN-driven and MRI-driven turbulence co-exist. This, however, seems unlikely, as SN activity has analytically been shown to suppress MRI in the inner 15 kpc region in a Milky Way like galaxy Korpi et al. (2010). MRI-driven turbulence and dynamos could, however, explain the unexpectedly large velocity dispersions in the extended HI discs of galaxies Sellwood and Balbus (1999), Tamburro et al. (2009).

A method suitable for numerically extracting the turbulent transport coefficients from anisotropic systems, such as galactic discs, which definitely have strong anisotropies arising from stratification, (non-uniform) rotation, and the presence of large-scale magnetic fields, was developed by Brandenburg and Sokoloff (2002). In such systems, $\alpha$ and $\beta$ are tensors instead of scalars. Thus, to extract all the tensor components, one needs many equations. Brandenburg and Sokoloff (2002) proposed that enough equations can be collected by forming various moments of the mean field components and their derivatives. Next, correlations with turbulent emf, again utilising the ansatz Eq. (11), are sought for. All of these quantities are measurable from a full MHD simulation. Their formulation of the resulting multidimensional regression problem was either spatially local, considering the value of the magnetic quantities and the turbulent emf at one single point only, or else non-local, considering a convolution kernel around the location. This method is referred to as the method of moments or the correlation method, and it has been extended to further improve the fitting procedure by using SVD (Simard et al. 2016; Bendre et al. 2020; Bendre and Subramanian 2022), which usually prevents unphysical solutions such as negative values of $\beta$. The method is known to work best for time-variable magnetic fields, and to underestimate the magnitude of $\beta$ especially for fields stable over time (Warnecke et al. 2018).

**Fig. 25:**
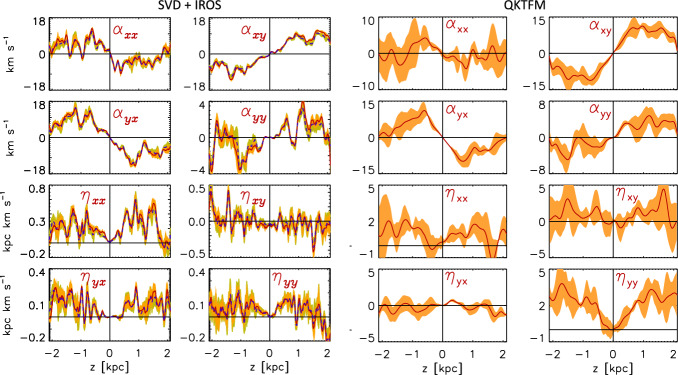
Derived turbulent transport coefficients from three different methods: *(left)* local SVD *(red, errors in orange)* and IROS *(blue dashed, errors in green)* results from Bendre et al. (2023); *(right)* QKTFM results from Bendre et al. (2020). The DNS-like MHD model from which these coefficients are derived is Model Q in Bendre et al. (2015), a local, Cartesian, SN-driven simulation with four times the rotation rate at the solar neighborhood and SN rate quater that of the Milky Way value.. The model develops large-scale dynamo action with oscillatory behavior in the kinematic stage, and dynamically significant, but stable in time, magnetic fields in the nonlinear stage. The turbulent transport coefficients are derived from the kinematic stage

An illustration of local SVD results from a local Cartesian MHD simulation with the NIRVANA code are shown in the leftmost set of Fig. 25, where components of the $\alpha$ tensor and the most relevant components of the turbulent resistivity tensor are shown as a function of the vertical height from the midplane. The overall sign convention and peak values of the $\alpha _{yy}$ component are rather similar to those found by Korpi (1999), although the profiles are quite complex and there are even sign reversals as functions of height, and strong gradients near the boundaries. The profiles of the diagonal turbulent resistivity tensor components are also complex, and unexpected negative values occur throughout the domain, especially near the top of it.

Recently, Bendre et al. (2023) developed a novel method, called iterative removal of sources (IROS), for extracting the turbulent transport coefficients. This method shares many similarities with the SVD method, while the main differences are the iterative fitting algorithm, which incrementally refines the estimates of the turbulent transport coefficients, and the possibility to define prior physical constraints (mIROS scheme). The latter property significantly decreases the errors, and the processing of extremely noisy data becomes possible. The first results of the IROS method are illustrated in Fig. 25 leftmost set, and compared to the SVD method. As is evident, these two methods give nearly identical results.

An important approach to measuring the turbulent transport coefficients is the TFM (Schrinner et al. 2005, 2007, recently reviewed in detail by Brandenburg 2018). In short, this method is based on the exertion of linearly independent test fields to the system that are passive in the sense of not interfering with it, but act as a diagnostic tool. The action of the turbulent flow on the test fields is monitored, and the equation for the fluctuating fields is directly solved for each test field. In the case of purely hydrodynamic turbulence present in the system, the method is said to work in the kinematic limit (KTFM). In magnetohydrodynamic simulations also magnetic fields can be generated by dynamos and tangling. In case large-scale magnetic fields and fluctuations produced from them by tangling are present, and they affect the flow field, the method works at the quasi-kinematic limit (QKTFM; retaining the KTFM formalism). If magnetic background turbulence, e.g., by SSD action, is present, then nonlinear extensions are required (see, e.g., Rheinhardt and Brandenburg 2010; Käpylä et al. 2022). Turbulent emf is approximated as local and instantaneous and its expansion is truncated after first order of the derivatives of $\overline{{{\varvec{B}}}}$ (Eq. (11)). This approximation is again used to solve for the turbulent transport coefficients. By directly solving for the fluctuating fields, many detrimental assumptions of the FOSA/SOCA are avoided. The local and instantaneous formulation imposes the restriction of capturing only effects of large-scale, slowly varying in time MFs. This restriction can, however, be relaxed by considering a formulation non-local in space and time (see, e.g., Rheinhardt and Brandenburg 2012). Typical results from QKTFM in the kinematic stage of a large-scale, dynamo-active, MHD model are shown in Fig. 25, rightmost set. Similar kinematic analysis on MHD models with varying parameters has been performed by Gressel et al. (2008, 2008) and Bendre et al. (2015).

In comparison to SVD, the profiles from QKTFM are overall somewhat smoother, while the correspondence of the magnitudes and vertical profiles of the $\alpha$ coefficients between the methods is satisfactory. The most notable difference is in the magnitude of the turbulent resistivity components. Both diagonal elements are roughly an order of magnitude larger with the QKTFM than with SVD. The off-diagonal component $\eta _{xy}$ is consistent with zero from SVD, while it has a clearly positive value from the QKTFM. On the other hand, $\eta _{yx}$ is consistently non-zero and positive from SVD, while consistent with zero from QKTFM. This does rule out the existence of the shear-current dynamo effect in galactic discs by both methods, the existence of which would require $\eta _{yx}$ being negative (see, e.g., Rogachevskii and Kleeorin 2003).

The quenching of the turbulent transport coefficients in the nonlinear regime, due to the presence of strong large-scale magnetic field, has also been studied with the QKTFM by Gressel et al. (2013a). Finally, Gressel and Elstner (2020) studied the importance of non-local and non-instantaneous effects in similar models, and found out that especially the former are important for galactic LSD. The $\alpha$ and $\eta$ coefficients were found to reduce when the scale of the test fields is made smaller, agreeing with results from simpler systems (e.g., Brandenburg et al. 2008). A surprising finding was that the turbulent pumping, generally directed towards the disc plane and aiding LSD, was becoming stronger with decreasing scale. This behavior is yet to be fully understood.

Until recently, DNS-like models to study galactic LSD have hovered at resolutions too coarse to properly capture SSD simultaneously. Such models are now emerging (Gent et al. 2024). In these models dynamically significant LSD is excited despite the co-existence of fast-growing SSD. This might not be so surprising, as an SSD alone in a multiphase, highly compressible fluid is weak, producing fluctuating fields with an energy of only a few percent of the kinetic energy in the turbulence (Gent et al. 2023). Also, these systems might exhibit much larger helicity fluxes than the simpler systems studied previously (see Sect. 4.2).

In this situation, determining the turbulent transport coefficients with the QKTFM is no longer straightforwardly possible, as the SSD generates background magnetic fluctuations that are not accounted for in the method. Viable extensions (Rheinhardt and Brandenburg 2010; Käpylä et al. 2020, 2022) have appeared, but even further extensions, such as relaxing isothermality, are required to deal with the galactic models. Furthermore, only the kinematic version of the compressible test-field method could be validated for very strong magnetic fields (Käpylä et al. 2022). Also, the required computational resources for the extraction of the turbulent transport coefficients significantly exceeds that for QKTFM. Evidently, SVD and IROS methods would not suffer from any of the discussed limitations, although the better noise-handling capacity of mIROS may turn out to be necessary.

Although the results of the different methods seem to be in rough agreement, it is still necessary to ask the question of which of the methods is the most reliable? The fitting-based methods SVD and IROS seem to agree well with each other, but they are both based on the use of the MF of the MHD simulation itself in the fitting procedure. In contrast, the test-field-based methods use a set of linearly independent and well defined test fields. In the fitting approach, the MF generated by the system could be degenerate, while the test-field approach guarantees that such a situation cannot occur, and the coefficients can be unambiguously determined. Also, the TFMs are rigorously tested against analytical solutions such as various incarnations of the Roberts flow (see, e.g., Rheinhardt and Brandenburg 2010), while such verifications have not been performed on the fitting-based methods. In Gressel and Elstner (2020) the authors propose that the scale-dependence of the transport coefficients could provide an explanation to the discrepancies between SVD and QKTFM, but this appears unlikely. Namely, the SVD approach uses the DNS-generated MF scale for the determination of both the $\alpha$ and $\eta$ coefficients, which both would become reduced, if the proposed explanation would be valid. Only the $\eta$ coefficients, however, show marked reduction.

The best way to test these discrepancies would be to compare the evolution of MF models using the measured coefficients to the actual MHD models. Curiously, this has been done with the SVD coefficients by Bendre et al. (2020), showing the two solutions to be in excellent agreement in the oscillatory kinematic regime. The period of dynamo cycles is known to directly depend on the turbulent resistivity (see, e.g., Shukurov and Subramanian 2021). Then one would expect to see a discrepancy between the QKTFM and MF results. This remains to be confirmed, and conclusions drawn accordingly.

The ultimate goal of the efforts to measure and describe the turbulent dynamo coefficients relevant for the LSD in galaxies is to build computationally more efficient global modelling frameworks. In essence, the goal is to build explicit large eddy simulations, where also the backscatter (or inverse cascade from small scales to larger ones) is captured in as much detail as possible. This has been attempted either in the MF framework (Gressel et al. 2013b) or in a simpler context for cosmological modelling (Liu et al. 2022). The most promising explicit large eddy frameworks exist only for simpler turbulence models (see, e.g., Grete et al. 2017).

### Measuring helicity fluxes

Helicity fluxes have also been measured from various types of MHD models. Such work is vital, as the final proof that helicity fluxes actually alleviate catastrophic quenching must come from full MHD models. The measurements necessarily involve many simulations with varying $\rm{Re}_{\rm{M}}$, reaching out to as high $\rm{Re}_{\rm{M}}$ as possible. This is demanding, so models remain limited to simplified, isotropically forced systems.

The rate of change of the mean helicity density of the small-scale field reads

25 $$\begin{aligned} \frac{\partial }{\partial t} \overline{{{\textbf {a}}}' \cdot {{\varvec{b}}'}} = -2 {{{\mathscr {E}}}}\cdot \overline{{{\varvec{B}}}}- 2 \eta \mu _0 \overline{{{\textbf {j}}}' \cdot {{\varvec{b}}'}} - \nabla \cdot \overline{{{\textbf {F}}}}, \end{aligned}$$

where the first term on the right hand side describes the generation of magnetic helicity and the second one its resistive destruction, while $\overline{{{\textbf {F}}}}$ is the small-scale helicity flux (see, e.g., Brandenburg and Subramanian 2005). If the flux is significant, its divergence should be dominant over the two other terms during a steady state dynamo. From the evolution equation for the dynamical $\alpha$ effect, Eq. (20), one would also expect that the mean field strength would become independent of $\rm{Re}_{\rm{M}}$, if the fluxes are efficient.

Del Sordo et al. (2013) studied an isothermal plane-wave forced Cartesian system with a possibility for an imposed wind in one coordinate direction. The forcing used was helical, producing an equator perpendicular to the wind direction by changing the sign of helicity across the equatorial plane. Although simplified, this model has some similarities to a local volume of a rotating galactic disc subject to a wind. The results show that the wind-like advection can induce a non-zero magnetic helicity flux, which shows asymptotic behavior as a function of $\rm{Re}_{\rm{M}}$. The other terms in Eq. (25) were larger for $\rm{Re}_{\rm{M}}< 1000$, but for higher $\rm{Re}_{\rm{M}}$ they became comparable to the divergence of helicity flux. Moreover, these terms showed an inverse dependence on $\rm{Re}_{\rm{M}}$. However, the MF decreased when the wind strength increased. Essentially similar results were obtained with a comparable but incompressible setup without a wind Rincon (2021). Agreeing with the results of Del Sordo et al. (2013) without the wind (Rincon 2021) found significant helicity fluxes due to the redistribution of helicity through turbulent flux across the equator. Although promising, these results do not yet conclusively prove that helicity fluxes can alleviate the catastrophic quenching scenario. Simulations with higher $\rm{Re}_{\rm{M}}$ and also including shear and more realistic turbulence driving are required.

## Conclusions

### Comparisons with analytic theory

Many aspects of analytic theory appear consistent with numerical simulations, in particular the existence of both SSD and LSD activity in galaxy models at different scales. SSD growth rates are strongly dependent on $\rm{Re}$ (Eq. 24), and thus on numerical resolution if it dominates resistivity over any physical resistivity implemented. The SSD and LSD compete for energy when both are active, but after the SSD saturates, the LSD continues to grow MF as predicted analytically (Sect. 3.3.3). The requirement of helical turbulence for dynamo activity is verified (Sect. 3.4.2). Parker instability, MBI, and MRI can drive further dynamo activity, although they are likely subdominant effects in star-forming regions of galactic disks (Sect. 3.3.3).

Turbulent transport coefficients derived from numerical models with multiple different sets of assumptions show that, although the overall picture of dynamo behaviour from analytic models does have some resemblance to the results, there are many inconsistencies (Sect. 4.1). These are most likely due to the strong, anisotropic, turbulent flows produced by the SN driving in stratified discs. As a result, simple dipolar or quadrupolar field configurations are likely to be more the exception than the rule (Sect. 3.4.2).

The question of the source of the magnetic fields that grow due to dynamo activity has had a major new mechanism proposed in recent years beyond classical battery processes. The new mechanism depends on the clear demonstration of dynamo action leading to near-equipartition fields occurring in plasmas magnetised *ab initio* by Weibel instability (Sect. 3.1). As this can occur already in protogalactic accretion flows, galaxies appear likely to be born magnetised. Fast SSD action in these high Re systems maintains fields within an order of magnitude of equipartition on less than megayear timescales. Large-scale MF does take much longer to develop, as it still relies on LSD activity over far longer timescales comparable to the rotational periods of galaxies (Sect. 3.3).

Many models suggest that helicity fluxes can prevent catastrophic quenching of LSD activity by small-scale magnetic fluctuations (Sect. 4.2). However, direct measurements of helicity fluxes in well-defined numerical experiments will still be required to conclusively demonstrate that catastrophic quenching does not occur in galactic dynamos.

### Consistency with observations

The observed global structure of magnetic fields has points of contact with models. The anticorrelation of magnetic spiral arms with stars and gas is sometimes seen in models including a circumgalactic medium (e.g., Steinwandel et al. 2019). This is consistent with results in the multiphase SSD, showing rapid SSD in the hot, diffuse ISM (Gent et al. 2023). Models show a wide variety of pitch angles, some of which do appear consistent with measurements of shallow negative values, although even positive values are seen in the models in perturbed or strongly interacting galaxies (Sect. 3.6).

Simulated maps of Faraday rotation derived from models agree best with observations if whole galaxy models are combined with a sub-grid model for Hii regions produced by stellar clusters drawn from a population synthesis model. High-galactic latitude regions in particular are dominated by structure within a few kiloparsecs of the Sun, if not the surface of the Local Bubble. Predictions of rotation measures are consistent with observed upper limits, but small enough that improved observations will be needed to constrain them (Sect. 3.6).

A clear definition of mean or regular fields needs to be given to adequately compare measurements by observation as outlined in Sect. 1.1 of ordered and turbulent fields, with averaging techniques typically employed in simulations and by theorists, such as horizontal averages. The choice of scales over which to define the regular field can be arbitrary, such as resolution beam size or the size of the numerical box, adding to the discrepency between observed and simulated quantities. While difficulty in satisfying the Reynolds rules arises from employing alternative averaging techniques, such as kernel smoothing or spectral filtering, it improves comparability with observations of field topology and ratios across various filtering scales. For example, applying measurements of the anisotropy of the random field for comparison with observations only makes sense if these arise via some form of filtering rather than extracting box or slice averages. As demonstrated by Gent et al. (2024) (see Fig. 8b), growth rates and ratios are not highly sensitive to the averaging technique, but it matters greatly to the apparent topology of the mean field.

### Challenges

Starting from the earliest times, models of seed field production and amplification by Weibel instability need to be extended towards realistic values of the electron-ion mass ratio, and checked for robustness against Landau damping. Quantitative demonstration of their applicability to protogalactic accretion flows will also be valuable. Increasing the domain size to further demonstrate growth up the turbulent cascade will begin to clarify whether this mechanism can indeed generate fields at astrophysically interesting scales.

However, the strong implication of these models that galaxies are born magnetised suggests that numerical models of galaxy formation and evolution should not start with infinitesimal fields, but rather with turbulent fields of some large fraction of equipartition, and that those minimum values of the turbulent field should be somehow maintained even if the numerical resolution of global or cosmological models is insufficient to directly capture the relevant fast SSD maintaining the field. This is necessary, because in comparison to galactic evolution timescales, the SSD grows almost instantaneously.

Current comparisons with observations suggest that the ratio of mean to turbulent field strength often disagrees with observations. Local models make too strong an MF, while many global models make too weak an MF. Resolving this contradiction will require work on both improved models and simulated observations. In order to understand global magnetic field properties, ultimately the combination of SSD and LSD must be simulated in a global context. This will first be possible for dwarf galaxies, where the necessary parsec-scale resolution can be reached in global models. Expanding such simulations to larger objects with well-developed disk structure will ultimately yield predictions of the large-scale MF and turbulent field structure. The use of uniform resolution grids will ensure that dynamo growth is not suppressed by numerical diffusion. However, low resolution results in slow SSD growth, suppressing strong mean field strengths until the SSD saturates. Both adaptive and unstructured mesh models need to be treated with great care, as they will indeed tend to have varying diffusion as their resolution varies.

To properly compare such predictions of global magnetic field properties to observations, simulated observations of the models will be required. Performing simulated observations of the synchrotron radiation and IR polarisation produced by the MF structure of galaxies in their cosmological context will require models of both magnetic field and cosmic ray evolution. These will allow much better constraints on the models to be placed by classical observations such as the pitch angle of spiral arms and field reversals.

Achieving sufficient resolution to measure dynamo coefficients in local models is a further challenge, that offers a potential solution to the challenge of global dynamo modeling. Such local models need to include the necessary physical processes in the ISM, including the formation of structures at larger than kiloparsec scales, such as superbubbles, galactic winds and fountains, and extraplanar ionized gas. To achieve this may require construction of reliable SGS models at the multi-parsec scale, possibly using machine learning techniques. If dynamo coefficients can be accurately and self-consistently measured in such models, they can be used to support the construction of explicit large eddy simulations that capture the sub-grid dynamo behavior.

Although preliminary work indicates agreement between MF models in some regimes and the DNS-like MHD models from which they draw their coefficients (Sect. 4.1), much further work needs to be done to extend this result into saturated regimes and to properly include the effects of SN driving of small-scale kinetic and magnetic energy. Disentangling the effects of multiple types of dynamos acting simultaneously remains a major challenge. Current SGS models only capture turbulent mixing, but not the inverse cascade from small-scale driving. Inclusion of SGS terms in the induction equation may be a direction to pursue to solve these problems.

## Footnotes

1 We note that if averaging not obeying the Reynolds rules would be used, Eq. (9) would have additional terms in ${\varvec{\mathscr{E}}}$.

2 L2 starts with a higher seed field to reduce the total integration time to saturation of the LSD.
